# Chemical Insights into Topical Agents in Intraocular Pressure Management: From Glaucoma Etiopathology to Therapeutic Approaches

**DOI:** 10.3390/pharmaceutics16020274

**Published:** 2024-02-15

**Authors:** Geewoo Nam Patton, Hyuck Jin Lee

**Affiliations:** Department of Chemistry Education, Kongju National University, Gongju 32588, Chungcheongnam-do, Republic of Korea; pgeewoo@gmail.com

**Keywords:** glaucoma etiopathology, intraocular pressure management, topical agent, pharmacology, clinical efficacy, glaucoma therapeutic approaches

## Abstract

Glaucoma encompasses a group of optic neuropathies characterized by complex and often elusive etiopathology, involvihttng neurodegeneration of the optic nerve in conjunction with abnormal intraocular pressure (IOP). Currently, there is no cure for glaucoma, and treatment strategies primarily aim to halt disease progression by managing IOP. This review delves into the etiopathology, diagnostic methods, and treatment approaches for glaucoma, with a special focus on IOP management. We discuss a range of active pharmaceutical ingredients used in glaucoma therapy, emphasizing their chemical structure, pharmacological action, therapeutic effectiveness, and safety/tolerability profiles. Notably, most of these therapeutic agents are administered as topical formulations, a critical aspect considering patient compliance and drug delivery efficiency. The classes of glaucoma therapeutics covered in this review include prostaglandin analogs, beta blockers, alpha agonists, carbonic anhydrase inhibitors, Rho kinase inhibitors, and miotic (cholinergic) agents. This comprehensive overview highlights the importance of topical administration in glaucoma treatment, offering insights into the current state and future directions of pharmacological management in glaucoma.

## 1. Introduction

Glaucoma is defined as a group of optic neuropathies characterized by the progressive degeneration of retinal ganglion cells (RGCs) of the optic nerve [1,2,3]. The global prevalence of glaucoma is estimated to reach 111 million by 2040 [4]. Such drastic epidemiological growth in the number of glaucoma patients is strongly correlated to the aging of the world population. Age is often considered a major risk factor for glaucoma based on its effect on the vulnerability of the optic nerve and efficiency in the systems involved in regulating aqueous humor outflow. The stealthy nature of glaucoma poses the greatest threat to the eye health of most glaucoma patients as early-stage glaucoma often progresses without noticeable symptoms. Substantial levels of neural damage (30–50% RGC degeneration) are usually discovered upon diagnosis following comprehensive examination and testing, which patients seek after noticing changes in their vision [5]. For these reasons, early detection of glaucoma is critical for preserving one’s visual field and preventing disease progression. These factors also implicate the possibility for low rates of diagnosis resulting in severe underestimates in the currently available epidemiological statistics.

Diagnosis of glaucoma is challenging due to its pathophysiological variability, lack of a standard reference for diagnosis, and issues stemming from the physical barriers of examining the eye (e.g., inaccurate IOP measurement and subjective evaluation of anatomical parameters). Therefore, longitudinal evaluation and thorough documentation are essential in the proper monitoring and diagnosis of glaucoma. Identification of patients with a high risk of developing glaucoma can serve as an effective approach for detecting the disease early. General risk factors of glaucoma include age, high myopia, diabetes, steroid medication (systemic or topical corticosteroids), prior eye injury, thin cornea, high blood pressure, heart disease, and sickle cell anemia [6]. Several genetic risk factors have been identified in previous studies (e.g., MYOC, PITX2, FOXC1, PAX6, CY1B1, LTBP2, OPTN, TBK1). A comprehensive review by Wang et al. presents the discovered glaucoma-associated genes in connection to various types of glaucoma and the biochemical and physiological influences of those gene mutations [7]. Despite the efforts to identify the genetic associations with glaucoma, our current understanding accounts for circa 10% of the total glaucoma cases worldwide [1]. A list of glaucoma-associated genes is presented in Table 1.

Our current comprehension of glaucoma etiopathology is centered around aberrant IOP in relation to neurodegeneration of the optic nerve. This notion, however, does not account for cases in which optic nerve damage is observed under normal IOP. Considering the current global prevalence and projections of its socioeconomic impact, glaucoma poses a progressively expanding detriment to the vision of the world population. Therefore, research efforts in advancing our understanding of the early stages of glaucoma and developing novel therapeutics that can effectively halt disease progression and possibly cure this disorder altogether are imperative in counteracting the increasing impact of the disease [8]. This comprehensive survey aims to provide an organized perspective on our current understanding of the following aspects of glaucoma: etiopathology, risk factors, methods of diagnosis, treatment approaches, and therapeutic agents.

## 2. Glaucoma Etiopathology

Responsible for most unilateral and bilateral blindness worldwide, glaucoma is defined as a classification of ocular disorders with two prevailing pathological features: abnormal intraocular pressure (IOP) and optic nerve damage [9]. With IOP anomalies as a central feature of glaucoma etiopathology, various types of the disease exhibit multifaceted pathogenesis intertwining structural (optic disk damage) and functional (visual impairment) pathologies [10]. In general, glaucomatous symptoms include elevated IOP, cupping (appearance of the optic disc), ocular pain, nausea, headaches, blurred vision, visual halos and glares, and vision loss (initiated at the midperiphery with centripetal progression) [1]. A key feature of the various types of glaucoma is the structural degradation of the optic nerve, responsible for transmitting visual information from the retina to the brain. Unlike other optic neuropathies, glaucoma patients exhibit optic nerve cupping, a phenomenon where the neuroretinal rim of the optic nerve becomes thinner and the optic nerve cup becomes larger due to retinal ganglion cell (RGC) loss [11]. The pathophysiological conditions that result in the surge of IOP inducing such pathogenic structural transformation vary substantially from one type of glaucoma to another [9]. A great deal of research has been dedicated to further our understanding of glaucoma etiopathology. Such endeavors have led to identifying pivotal physiological and clinical aspects of these ocular disorders and developing various therapeutics; however, the exact biochemical mechanisms through which glaucoma is developed are only beginning to be understood [12]. For instance, the mechanistic causative factors in the most common form of glaucoma, primary open angle glaucoma (POAG), remain elusive. Identification of such fundamental biochemical components responsible for glaucomatous neurodegeneration could be exceedingly beneficial in the early diagnosis of disease and development of specified treatment approaches with minimal side effects.

### 2.1. Types of Glaucoma

Types of glaucoma (Table 1) can be first organized as open angle or angle closure glaucoma based on the underlying ocular anatomical configuration of the aqueous humor outflow pathway [13]. Then, they can be further divided into primary and secondary glaucoma. Glaucoma is considered primary if no preexisting ocular conditions, such as injury or disease, are present. Secondary glaucoma is caused by external factors including ocular injuries, disorders, and medications. Primary glaucomas include POAG, primary angle closure glaucoma (PACG), normal tension glaucoma (NTG), and primary congenital glaucoma (PCG). Secondary glaucomas include neovascular glaucoma, pigmentary glaucoma, exfoliative glaucoma, and uveitic glaucoma. Except for NTG, all forms of glaucoma exhibit aberrant changes in IOP that lead to optic nerve damage and, ultimately, visual impairments [14]. In this section, the current understanding of the different glaucoma types will be briefly discussed.

The most common form of glaucoma, POAG, is responsible for 12.3% of blindness worldwide with a 1.86% prevalence in people older than 40 years of age [15]. The majority of POAG cases are reported in patients of European and African descent [13]. In 2016, POAG was projected to cause 6 million cases of blindness worldwide by the year 2020 [13]. POAG can occur with or without elevated IOP, with the latter categorized as normal tension glaucoma (NTG), as discussed below. Depending on the onset of POAG, it can be further separated into adult-onset (after 40 years of age) and juvenile-onset (3–40 years old) [11]. The early development of POAG is gradual and often undetected until noticeable visual field loss has occurred [13]. Risk factors for POAG include age, male sex, high IOP, high myopia, hypertension, type 2 diabetes mellitus, low cerebral fluid pressure, and genetic history of POAG [13,16]. The defining characteristics of POAG are the deficiencies in aqueous humor drainage by the trabecular meshwork, which leads to the accumulation of aqueous humor within the eye and, subsequently, increased IOP, RGC loss, optic nerve damage, and visual deficiencies [17]. The clinical definition of POAG does not include the elevation of IOP as it can occur in the normal range of IOP (circa 10–21 mmHg) [11]. Regardless, IOP is considered a causative factor in POAG and its only modifiable therapeutic target [18]. Augmented IOP exhibits direct physical and biochemical stress on RGCs: (i) decreased axonal transport leading to the nutritional deprivation of neurotrophic factors, (ii) tissue hypoxia at the optic nerve head resulting in cellular oxidative stress, (iii) glial cell activation inducing the degradation and remodeling of extracellular matrix invoking biomechanical stress on RGCs, and (iv) TNF-α-mediated RGC damage [19,20,21,22]. Clinical studies implicate lamina cribrosa, a collagenous structure through which the optic nerve exits the eye, as the initial location of glaucomatous nerve damage [23]. Kwon et al. suggest that cell-mediated mechanisms potentially involving excessive synthesis of extracellular matrix materials or increased intra-axonal Ca(II) levels from the overexpression of ephrin-B2 could cause neurodegeneration of RGC axons [11]. RGC degradation is followed by a neuroinflammatory scar response via glial activity by expressing major histocompatibility complex class II molecules and components of the complement cascade that could exacerbate the pathogenic conditions and further promote RGC loss in POAG [24,25].

The second most common form of glaucoma is PACG, globally accountable for about half of glaucoma-related blindness. In 2020, the global prevalence of PACG was reported at 0.6%, affecting circa 17.1 million people in the world, with a dominant portion of those patients being Asian females [26]. PACG is characterized by the mechanical obstruction of the trabecular meshwork due to pathological changes in ocular anatomy involving the appositional approximation between the iris and trabecular meshwork or lens. These pathogenic circumstances denote the narrowing of the anterior chamber angle that prompts the blockage of aqueous humor outflow and increase in IOP [27,28]. Anatomical risk factors of PACG include small cornea, shallow anterior chamber, thick lens, anterior lens position, and short axial length [29,30]. PACG can fall under various classifications depending on the clinical symptoms, history, anatomy, and etiology. Temporal aspects of PACG pathology present two categorizations: acute and chronic. Acute PACG is often accompanied by a quick and complete obstruction of the trabecular meshwork, posing a medical emergency, during which spikes in IOP can damage the optic nerve [27]. The lack of timely medical intervention of acute PACG can lead to significant damage, visual impairment, and transition to its chronic form [28]. On the other hand, chronic PACG develops more gradually, making its detection more difficult, like POAG. PACG is divided into three stages: (i) primary angle closure suspect, representing the first stage, where reversible contact between the peripheral iris and trabecular meshwork is present, and IOP is normal, (ii) primary angle closure is marked by elevated IOP, but without notable glaucomatous optic neuropathy, and (iii) lastly, primary angle closure glaucoma is the final stage in which glaucomatous optic neuropathy is evident [31,32]. The pathogenic classification of PACG considers an intertwined system of four anatomical features leading to a narrow anterior chamber angle and crowded anterior segment: iris, ciliary body, lens, and vectors posterior to the lens [27]. Classical mechanisms of the anterior chamber angle closure in PACG can be divided into two types: pupillary and non-pupillary block [28]. Pupillary block, which can be caused by antitussive or nasal decongestants, describes the increasing proximity between the lens and the posterior face of the iris [33,34]. The latter denotes a thick peripheral iris, anterior location of the iris, anterior rotation of the ciliary body, and plateau iris. Many PACG cases manifest a multi-mechanistic pathogenesis leading to elevated IOP and optic nerve damage [35]. Associations have previously been made between the IOP-induced optic neuropathy of PACG and that of POAG; however, reports suggest that the PACG-induced optic nerve damage exhibits a distinct mechanism from POAG, as evident by differences in the pathological changes in the optic nerve head [36,37,38]. Sun et al. state that PACG is also a psychosomatic disease with a more complex etiopathology with potential psychological and neural effects [28]. Treatment methods for PACG often focus on addressing the blockage of the aqueous humor outflow passage through the broadening of the anterior chamber angle or provision of alternative outflow pathways to control the IOP, as presented below in the glaucoma therapeutics sections [28].

Normal tension glaucoma (NTG) signifies an exemption of glaucomatous optic neuropathy surrounding elevated IOP in that this ocular disorder is not marked by a notable increase in IOP [39]. The fact that NTG displays the typical glaucomatous disk changes, visual field defects, and open anterior chamber angles at relatively normal IOP (<21 mmHg) raises numerous questions regarding the true pathological implications of elevated IOP [40]. Killer et al. suggest that although pressure is a scalar force that is applied homogeneously throughout the eye, optic nerve damage does not occur homogeneously, implicating the potential for alternative pathogenic pathways of neurodegeneration [39]. Research endeavors striving to identify such causative factors of NTG have led to the introduction of various theories encompassing heightened cellular and histochemical sensitivity to IOP, local or generalized vascular dysregulation, elevated IOP gradient across the lamina cribrosa, and cerebrospinal circulation impairment [39]. Recent studies propose that chronic low vascular perfusion, Raynaud’s phenomenon, migraine, nocturnal systemic hypotension, head down yoga, and over-treated systemic hypertension are the main causes of NTG [40,41]. An interesting aspect of NTG is that, despite moderate IOP, its treatment focuses on the reduction in IOP by circa 30% with demonstrated clinical efficacy [42]. Such findings implicate the possibility for significant interindividual variability in the sensitivity of the optic nerve against IOP, where patients with NTG exhibit a greater optic nerve sensitivity that renders it more susceptible to neurodegeneration under smaller changes in IOP [39].

PCG is a developmental nonsyndromic glaucoma in infancy, before the age of three, that can lead to blindness at early stages of life [43]. Studies have discovered that PCG is more prevalent in populations with a higher prevalence of consanguinity and is associated with CYP1B1 gene mutations which show variable expressivity and phenotypes [43]. Physiological symptoms of PCG include elevated IOP, globe enlargement, edema, anterior sclera thinning, iris atrophy, opacification of the cornea with Descemet’s membrane rupture, and progressive glaucomatous optic atrophy [44]. Furthermore, progressive reduction in visual acuity and loss of visual fields ultimately lead to invariable blindness without proper treatment [45]. In 1875, angle structure malformation in children was claimed as the culprit for PCG, with the disorder being considered untreatable until 1938, when goniotomy was introduced [46]. In comparison with other forms of glaucoma, PCG diagnosis is considered relatively simple. The presence of the three classical features of PCG (epiphora, excessive tearing; photophobia, sensitivity to light; and blepharospasm, uncontrollable blinking) leads to the rapid diagnosis of the disorder. The arrested development of neural crest tissue, a collection of multipotent stem cells located at the side of the neural tube proximal to the epidermal layer after neurulation, often causes an immature angle appearance in PCG [43,47]. These early-stage structural abnormalities lead to impaired outflow of aqueous humor, resulting in the increase in IOP and optic nerve damage. Due to the infantile nature of PCG, early detection is challenging but critical for preserving vision in children. Surgical measures (e.g., goniotomy, trabeculotomy, and trabeculectomy) addressing angle closure by reducing resistance through internal or external approaches are considered the first-line treatment for PCG [48]. Internal therapeutic operations in PCG patients are applied when mild or moderate corneal edema is present, while patients with substantial corneal clouding are treated with external measures such as trabeculotomy or trabeculectomy [46].

Secondary glaucoma represents a group of glaucoma that develops as a consequence of other underlying ocular or systemic conditions [49]. Unlike primary glaucoma, where the cause of increased IOP is not immediately linked to another condition, secondary glaucoma occurs due to identifiable factors such as neovascularization, iris pigment, excessive extracellular debris, inflammation (uveitis), intraocular tumors, use of corticosteroid eye drops or systemic steroids, systemic disease, periocular steroid cream, iridocorneal endothelial syndrome, angle recession, epithelial downgrowth, and the use of systemic medications [50,51]. These factors can disrupt the normal flow of aqueous humor, leading to elevated IOP and potential damage to the optic nerve. Secondary glaucoma can develop suddenly or gradually, depending on the underlying cause. Neovascular glaucoma is characterized by the neovascularization of the iris [52]. The formation of new vessels over the iris and the iridocorneal angle can lead to an increase in resistance against aqueous humor outflow, leading to elevated IOP. This phenomenon is often a secondary consequence of proliferative diabetic retinopathy or central vein retinal occlusion [52]. Pathogenic angiogenesis is driven by disruption in the homeostasis of pro- and anti-angiogenic factors [e.g., vascular endothelial growth factor (VEGF), pigment epithelium-derived factors (PEDF), and basic fibroblast growth factor (bFGF)] [53,54]. Pigmentary glaucoma is a specific form of glaucoma induced by the dispersion of pigment granules from the iris into the eye’s drainage structures [55]. These pigment granules can accumulate and obstruct the trabecular meshwork, leading to a reduction in aqueous outflow and an increase in IOP [56]. This condition can be triggered by activities that cause the iris to rub against the lens, releasing pigment particles into the aqueous humor [55]. Exfoliative glaucoma is a distinct form of glaucoma characterized by the accumulation of abnormal amounts of extracellular material on various structures within the eye. Excessive buildup of extracellular materials in the lens, iris, and other parts of the anterior segment can compromise normal drainage pathways for aqueous humor, causing an increase in IOP [57]. Glaucoma research efforts have led to the identification and categorization of numerous types of ocular disorders. Although manifesting different pathological pathways to optic nerve damage, elevation of IOP can be observed as a prevalent feature of this group of diseases. Therefore, imbalances between the production and outflow of aqueous humor in the eye, abnormal IOP, optic neurodegeneration, and, ultimately, loss of vision are considered the key pathological qualities of glaucoma. Hence, the treatment of the various glaucomas tends to focus on managing such facets of this globally prevalent ocular condition.

### 2.2. Risk Factors

Numerous risk factors have been associated with the development of various types of glaucoma. These include elevated IOP, age, family history of glaucoma, ethnicity, thin cornea, preexisting eye and systemic health conditions, use of corticosteroids, personal eye anatomy, and sedentary and unhealthy lifestyle features [7]. More specifically, the risk factors for POAG are elevated IOP, age, familial history, African ancestry, myopia, and presence of systemic diseases; PACG risk factors are hyperopia, familial history, age, female gender, Asian or Inuit descent, shallow anterior chamber depth, shorter axial length, and thicker lens; NTG risk factors include optic nerve and anatomical anomalies, elevated IOP, systemic vascular disease; pigmentary glaucoma risk factors comprise myopia, flat cornea, familial history, age, male gender, and concave iris and posterior iris insertion; risk factors of exfoliative glaucoma are age and Scandinavian and Mediterranean race [4,29,56,57,58].

As discussed above, elevated IOP is often seen as the primary evidence and causative factor in the pathogenesis of glaucoma; this aspect of the disease, however, is not absolute, as patients without abnormally high IOP can exhibit significant glaucoma-induced visual, field loss as observed in NTG [59]. Such features of the disease are puzzling and portray the complex and multifactorial nature of its etiopathology. Strong correlations have been reported between age and glaucoma onset, where significant increases in glaucoma incidence can be observed with increasing age [60,61,62]. Optical changes in the aqueous humor dynamics caused by decreases in outflow facility from the accumulation of extracellular material in both the ciliary muscle and trabecular meshwork along with the loss of trabecular meshwork cells have been implicated as an age-related causative pathway leading to aqueous humor accrual and elevated IOP [63]. An age-associated decrease in the physiological and optical levels of hyaluronic acid is indicated to result in the increased expression of fibronectin and thrombospondin, which in turn can invoke a cyclic negative pathway involving the production of transforming growth factor β, interleukin-1, and CD44S, that ultimately exacerbates the buildup of extracellular matrix material, further contributing to the pathogenic pathways of glaucoma [64]. Patients with first-degree relatives diagnosed with glaucoma are at increased risk of developing the disease. The National Eye Institute recommends that high-risk glaucoma patients receive comprehensive dilated eye exams once every two years. These groups include African Americans over age 40; everyone over age 60, especially Mexican Americans; and people with a family history of the disease. Although glaucoma can transpire at all ages, early-onset (before age 40) exhibits Mendelian inheritance and adult-onset forms (after age 40) can be inherited as complex traits [65,66]. Recent research has made connections between early-onset glaucoma and MYOC, OPTN, and TBK1 mutations [65]. Other genes have been linked to POAG (ABCA1, AFAP1, GMDS, PMM2, TGFBR3, FNDC3B, ARHGEF12, GAS7, FOXC1, ATXN2, and TXNRD2) and PACG (DR1, CHAT, GLIS3, FERMT2, and DPM2-FAM102) [65].

### 2.3. Aqueous Humor Dynamics

Located in front of the lens, the aqueous humor is a transparent liquid composed of organic and inorganic ions, glutathione, carbohydrates, amino acids, carbon dioxide, oxygen, and water. This slightly alkaline ocular fluid is responsible for various physiological functions within the eye. Its functional roles include nutritional supply to the avascular tissues of the cornea and lens, waste product removal, maintenance of the shape and structure of the eye by exerting adequate IOP against the walls of the eye, contribution in focusing light based on its refractive index, and protection against external forces and injuries by acting as a physical cushion. The balance between the production/secretion and outflow of aqueous humor is directly linked to IOP maintenance [67]. Three mechanisms are involved in aqueous humor formation: diffusion, ultrafiltration, and active secretion. Active cellular secretion by the ciliary epithelium is a major contributor in producing aqueous humor at a flow rate of circa 2–3 µL/min [68]. Within the ciliary body, a region called pars plicata is where the ciliary processes, sites of aqueous humor production, can be found. Once secreted, the aqueous humor flows into the space between the lens and the iris, the posterior chamber, and then through the pupil into the front part of the eye, the anterior chamber, where a temperature gradient creates a convective flow pattern [67]. It should be noted that the circadian rhythm has a prominent impact on aqueous humor flow, which is higher in the morning than at night [68].

**Figure 1:**
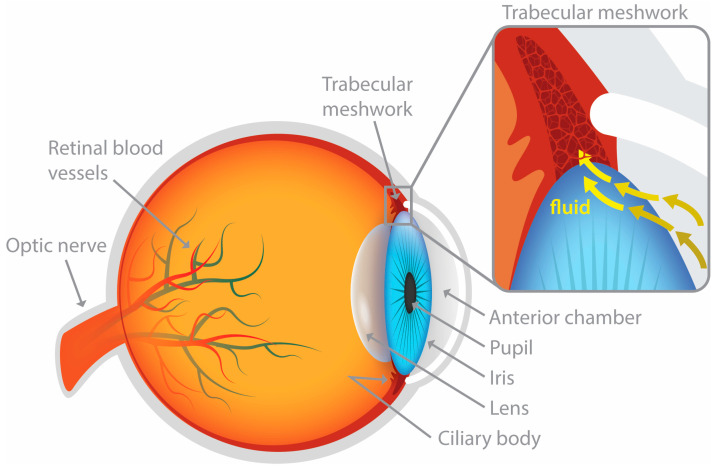
Conventional (trabecular meshwork) pathway of aqueous humor outflow.

Aqueous humor outflow is accomplished through two passive flow pathways: the trabecular meshwork (conventional; Figure 1) and the uveoscleral (unconventional) pathway [64]. Located near the front of the eye at the junction between the iris and the cornea, the trabecular meshwork is a specialized tissue structure consisting of a network of fine, sieve-like beams and sheets that plays a critical role in regulating the drainage of the aqueous humor [69]. This net of tissue acts as a filter allowing the aqueous humor to exit the anterior chamber. After passing through the trabecular meshwork, the aqueous humor flows into a channel called Schlemm’s canal, which is located near the outer edge of the cornea. From Schlemm’s canal, the aqueous humor drains into larger vessels (e.g., intrascleral channels) and then is absorbed by the bloodstream at the episcleral and conjunctival veins [70]. The conventional outflow pathway accounts for approximately 70–95% of the aqueous humor outflow [71]. Unlike the conventional trabecular outflow pathway, the unconventional outflow pathway or the uveoscleral outflow pathway is not a distinctive pathway with tubes and channels [72]. Studies insinuate the secondary role of the unconventional pathway through which additional aqueous humor can be released from the anterior chamber when trabecular resistance emerges [72]. The uveoscleral outflow pathway is a less defined route through which aqueous humor trickles through, around, and between tissues, including the supraciliary space, ciliary muscle, suprachoroidal space, choroidal vessels, emissary canals, sclera, and lymphatic vessels [73]. This ambiguity has led to the label of ‘unconventional outflow pathway.’ Estimates suggest that 5–30% of the total aqueous humor outflow can be accounted for by the unconventional pathway [74]. Notable interest in the uveoscleral pathway emerged with the discovery that prostaglandin analogs could relieve elevated IOP by increasing the magnitude of its flow [75].

Based on our current understanding of glaucoma etiopathology encompassing the central notion of IOP elevation, the balance in the production and elimination of aqueous humor presents itself as an essential component of the disease regardless of its specific categorization. These central ideas have led to innumerable research endeavors to identify the pathogenic factors influencing this delicate equilibrium. Furthermore, the currently available treatments aim to reduce IOP by either reducing aqueous humor production or increasing aqueous humor outflow. Glaucoma therapeutics aim to stop the progression of the disease and vision loss; there is no cure available. Therefore, early detection and diagnosis of glaucoma is crucial in preventing visual field loss. Due to the surreptitious nature of these diseases, early detection can be extremely challenging as the initial development and onset of vision loss may be inconspicuous.

## 3. Diagnosis of Glaucoma

Glaucoma diagnosis presents major challenges due to the lack of standardized guidelines and difficulties in accurately making measurements within the eye. Early stages of glaucoma are asymptomatic and are often undetected, which can result in irreversible visual impairments before patients recognize changes in their vision [76]. Substantial variability in the symptoms and pathophysiological effects of the disease further complicates the matter for ophthalmologists, as certain patients may exhibit detrimental conditions associated with glaucoma onset and vision loss without the onset of the disease and vice versa. For example, substantial variance can be observed not only from patient to patient, but also from the same patient depending on the time of day. For these reasons, longitudinal evaluation and documentation are critical in effectively monitoring and accurately diagnosing glaucoma. This also makes the early detection of glaucoma exceptionally difficult. Glaucoma diagnostic procedures often consist of detailed examinations of the following parameters: medical, family, and social history, IOP, anterior chamber angle, corneal thickness, optic nerve, nerve fiber layer, and visual field [77]. In this section, a brief overview of the processes that lead to the diagnosis of glaucoma and the obstacles to such efforts will be provided.

### 3.1. Tonometry

Considering the central role of IOP in glaucoma etiopathology, tonometry is the first line of testing when potential ocular damage is suspected from glaucoma. Ocular tonometry is used to measure IOP, defined as the fluid pressure of the eye. Simply put, IOP is a measure of the force applied by the aqueous humor on the internal surface of the anterior eye [78]. Two main types of tonometry are applanation (Goldmann and non-contact) and indentation (Schiotz tonometry, Pneumotonometry, Tono-pen) tonometry. Other techniques include rebound and Pascal dynamic contour tonometry. It is common practice to take multiple measures of IOP using several distinct methods to obtain a more precise measurement through comparative evaluation of various IOP measurements. 

Applanation tonometry determines IOP by directly applying force onto the corneal surface until it is flattened; the applanating force or the area flattened is used to calculate the IOP [79]. These estimations are based on the Imbert-Fick principle, proclaiming that the pressure inside an ideal, dry, infinitely thin sphere with an elastic membrane wall equals the force necessary to flatten its surface divided by the area of flattening (P = F/A, where P = pressure, F = force and A = area) [79,80]. Considered the gold standard IOP test, Goldmann applanation tonometry quantifies the force necessary to flatten a corneal area of 3.06 mm diameter [81]. Before measurement, a fluorescein dye is administered to the patient’s eye to highlight the tear film [79]. Subsequently, a split-image prism is used to divide the image of the tear meniscus into a superior and an inferior arc; IOP is obtained when these arcs are aligned such that their inner margins touch. Measured in mmHg, IOP is equal to the flattening force (g) multiplied by 10. Potential causes for inaccuracy in Goldmann applanation tonometry include the following: insufficient or excessive fluorescein, high astigmatism, irregular or scarred cornea, and abnormal corneal thickness [81]. The Perkins tonometer is a portable Goldmann applanation tonometer. 

Non-contact tonometry, air puff tonometry, and an ocular response analyzer employ an air column of increasing intensity to flatten the cornea [82]. The air column is stopped when the cornea is flattened and the corresponding force is used to calculate IOP in mmHg. The air puff tonometer reportedly underestimates high IOP and overestimates low IOP, when compared to the measurements of Goldmann applanation tonometry [83]. In contrast to the air puff tonometer, the ocular response analyzer takes into account the elasticity of the ocular applanation point [84]. The air column is applied and the point at which applanation occurs is noted; thereafter, the air column persists until the cornea is indented [85]. At this time, the intensity of the air column is gradually decreased until applanation. The two points of data regarding applanation in both directions are used to estimate the IOP [85]. 

Indentation tonometry engages the principle that a predetermined force will indent a soft eye further than a hard eye [85]. The Schiotz tonometer places a footplate attached to a weighted plunger onto the cornea. The magnitude of how much the plunger sinks into the cornea is read at the scale at the top of the plunger. This number is then converted to IOP using a conversion table [86]. 

The pneumotonometer combines features of both applanation tonometry and indentation tonometry. It is equipped with a moderately convex silicone tip, measuring 5 mm in diameter, situated at the tip of a piston hovering on a stream of air. This silicone tip gently indents the cornea. As the cornea and the tip align flatly, the pressure exerted on the tip becomes equivalent to the IOP. At this juncture, the device records the pressure within the system, presenting the result in mmHg. These measurements exhibit a strong correlation with those obtained through Goldmann applanation tonometry, particularly within the normal IOP range [87]. 

The Tono-pen also combines aspects of applanation and indentation techniques. A compact, handheld, battery-operated, portable device, the Tono-pen is equipped with an applanating footplate featuring a small, minimally protruding plunger at its center [88]. Upon contact with the eye, the plunger encounters resistance from the cornea, resulting in a gradual increase in force that is measured by a strain gauge [88]. At the precise moment of applanation, the force is evenly distributed between the footplate and the plunger, causing a brief, slight reduction in the continuously rising force [89]. This particular point of applanation is electronically recorded. Multiple measurements are averaged, and since the applanated area is known, the IOP can be calculated accordingly [87]. These measurements demonstrate a strong correlation with Goldmann tonometry, particularly within the normal range of IOP values. 

Several reviews comprehensively investigate the accuracy and precision of the various tonometry techniques mentioned above [81,90]. Research regarding the effect of central corneal thickness (CCT) has shown that thicker CCT may lead to an artificially high IOP measurement, whereas thinner CCT can lead to underestimates [91]. Furthermore, Ko et al. experimentally demonstrated that Goldmann applanation tonometry was subject to the least amount of CCT-related measurement variance, while non-contact tonometry was the most affected by CCT [91]. Due to the possible effects of CCT on tonometry measurements, CCT measurements have become a part of a complete ophthalmic examination in suspected glaucoma patients [92,93]. 

### 3.2. Gonioscopy

Gonioscopy is an ocular exam technique capable of visualizing the anterior chamber angle, which contains the trabecular meshwork, located between the cornea and iris [94]. An excessively narrow anterior chamber angle is a key pathogenic feature of PACG. This angle cannot be observed directly due to total internal reflection at the tear-air interface. However, the utilization of a contact gonioscopy lens can overcome this limitation by permitting light to penetrate the tear–air interface and subsequently reflect off a mirror back to the examiner’s eye [94]. This unique approach enables clinicians to visualize the anterior chamber angle, providing them with essential diagnostic insight.

### 3.3. Optic Nerve Assessment

**Figure 2:**
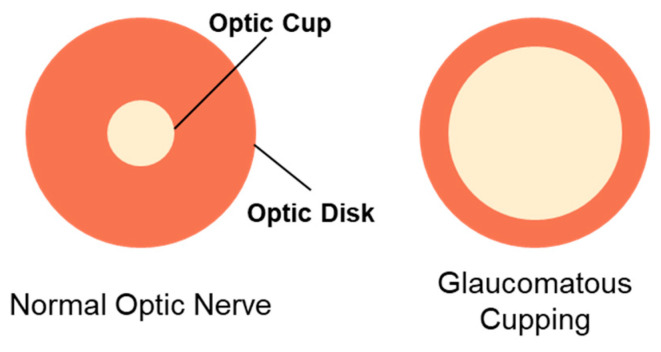
A graphical representation of optic nerve cupping. The inner and outer circles represent the optic cup and optic disk, respectively. Notable increases in the ratio of cup-to-disk diameter indicate glaucomatous cupping.

Responsible for relaying visual information from the eye to the brain, the optic disk of the optic nerve comprises circa 1.2 million ganglion cells lining the inner retina [95]. Research has discovered the various structural changes induced by the neuropathic pathology of glaucoma [96]. Strong correlations have been reported between the detection of apparent anomalies in the optic nerve and visual field loss in glaucoma [97]. Conspicuous changes in the visual appearance of the optic nerve, therefore, are considered a hallmark in glaucoma, with implications in the state and severity of the disease [98]. The optic nerve is approximately 3 mm in diameter, but shows significant interindividual variability [96,99]. Comprehensive assessment of the optic nerve is a critical part of evaluating the extent of glaucoma-associated neuropathy. The advancement of technology concerning optic nerve imaging has enabled healthcare providers to monitor the optic nerve more precisely. Optic nerve assessment employs numerous steps and a combination of methods. First, a physical examination of the optic nerve is performed with a silt lamp or direct ophthalmoscope to assess optic nerve cupping, a key pathological event of glaucoma where the cup-to-disk ratio is augmented (Figure 2). Optic nerve cupping consists of two main components, prelaminar and laminar thinning. The former is associated with retinal ganglion cell loss, while the latter is indicative of damage to the lamina cribrosa and peripapillary scleral connective tissue [100]. Despite its strong connection to glaucoma, the presence of cupping does not directly dictate disease onset and progression. Therefore, differentiating between glaucomatous and non-glaucomatous optic nerve cupping is important, but challenging [100]. Numerous aspects of the optic nerve cup are examined during the physical examination, including (i) size and shape of the optic disk, (ii) size, shape, and pallor of the neuroretinal rim, (iii) size of the optic cup in relation to the area of the disk, (iv) configuration and depth of the optic cup, (v) ratios of cup-to-disk diameter and cup-to-disk area, (vi) position of the exit of the central retinal vessel trunk on the lamina cribrosa surface, (vii) presence and location of splinter-shaped hemorrhages, (viii) occurrence, size, configuration, and location of parapapillary chorioretinal atrophy, (ix) diffuse and/or focal decrease of the diameter of the retinal arterioles, and (x) visibility of the retinal nerve fiber layer (RNFL) [96].

Following the physical examination of the optic nerve, additional imaging methods are employed to further inspect the optic disk through techniques such as optic disk stereophotography, optical coherence tomography (OCT), confocal scanning laser ophthalmoscopy (CSLO), or Heidelberg retina tomography (HRT), and scanning laser polarimetry (SLP). Optic disk stereophotography involves the imaging of the optic disk from different angles to create a stereo or 3D effect when the images are viewed together, to provide a better perspective of the optic disk enabling the perception of depth [101]. OCT is capable of obtaining high-resolution cross-sectional images of the optic nerve head, including the optic disk and neuroretinal rim, to monitor the structural changes and measure various pathogenic parameters of glaucoma, including RNFL thickness, ganglion cell layer thickness, cup-to-disk ratio, and the size, shape, and pallor of the neuroretinal rim [102]. CSLO utilizes a diode laser (670 nm) to scan the retinal surface and image the optic nerve head and RNFL. This method provides both quantitative and qualitative data regarding the optic nerve head and RNFL. Numerical values for the following parameters are provided: disc area, cup area, rim area, cup volume, rim volume, cup/disk area ratio, linear cup/disk ratio, mean cup depth, maximum cup depth, cup shape measure, height variation contour, mean RNFL thickness, and RNFL cross-sectional area [103]. Recent developments in the CSLO software (HRT III, software version 3) provide statistical analysis and prediction based on the obtained data in the form of a glaucoma probability score [104]. SLP provides RNFL thickness measurements based on the birefringence of the retinal ganglion cell axons [105]. The abovementioned imaging techniques allow ophthalmologists to meticulously inspect the optic nerve to effectively and accurately diagnose glaucoma through qualitative analysis, significantly improving their capacity for early detection.

### 3.4. Visual Field Test

The visual field test, also known as perimetry, is a fundamental diagnostic tool for glaucoma that assesses a patient’s peripheral and central vision by measuring their ability to perceive visual stimuli at various locations within their visual field [106]. Modern versions of the test utilize automated perimeters, such as the Humphrey Visual Field Analyzer, which employs computerized algorithms to precisely map a patient’s visual field. In the context of glaucoma, the visual field test plays a pivotal role in both diagnosis and treatment monitoring. It helps clinicians detect and characterize glaucomatous visual field defects, enabling early diagnosis. Moreover, during treatment, regular visual field tests track the progression of glaucomatous damage and the effectiveness of therapeutic interventions. These quantitative data inform clinical decisions, allowing for timely adjustments to treatment plans and ensuring the preservation of the patient’s visual function and quality of life.

Diagnosis of glaucoma is a cornerstone in the comprehensive management of this sight-threatening condition, with early detection being critical in minimizing visual field defects. Our exploration of the glaucoma etiopathology has underscored the multifactorial nature of the disease, where both intraocular pressure and various structural and vascular factors converge to initiate and perpetuate optic nerve damage. However, the evolving landscape of diagnostic modalities, ranging from advanced imaging techniques like OCT and CSLO to functional assessments such as visual field testing, empowers clinicians to make earlier and more accurate diagnoses. The integration of these modalities has not only enhanced our understanding of the disease but has also provided a solid foundation for personalized treatment strategies tailored to the unique characteristics of each patient. Moving forward, as our understanding of glaucoma’s complex etiology continues to deepen, the quest for innovative therapeutics remains ever more promising, aiming not only to halt disease progression but to improve the quality of life for those living with glaucoma.

## 4. Glaucoma Therapeutics

**Figure 3:**
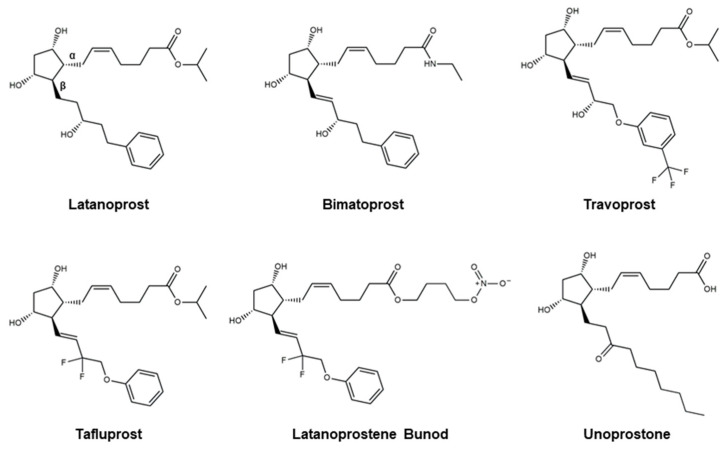
Chemical structures of six prostaglandin analogs with reported clinical efficacy in managing intraocular pressure (IOP) for the treatment of glaucoma.

**Figure 4:**
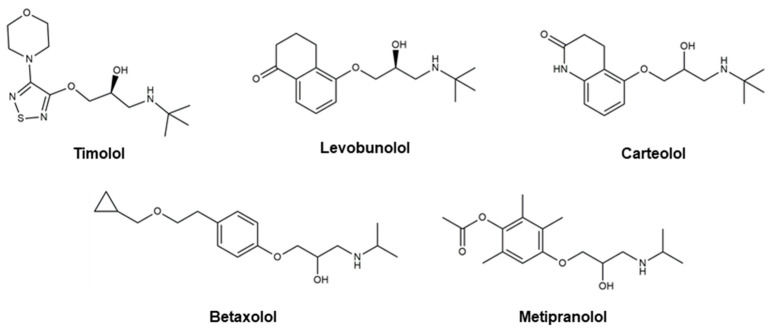
Chemical structures of five beta blockers with reported clinical efficacy in managing intraocular pressure (IOP) for the treatment of glaucoma.

**Figure 5:**
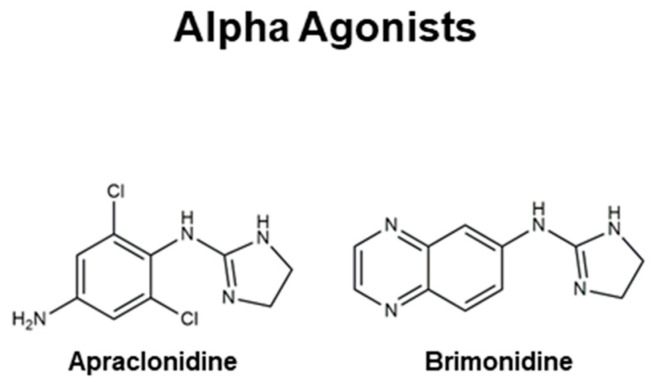
Molecular structures of two alpha agonists with reported clinical efficacy in managing intra-ocular pressure (IOP) for the treatment of glaucoma.

**Figure 6:**
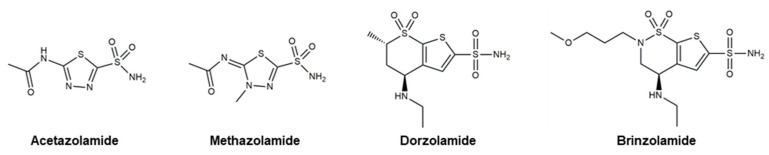
Chemical structures of four carbonic anhydrase inhibitors with reported clinical efficacy in managing intraocular pressure (IOP) for the treatment of glaucoma.

**Figure 7:**
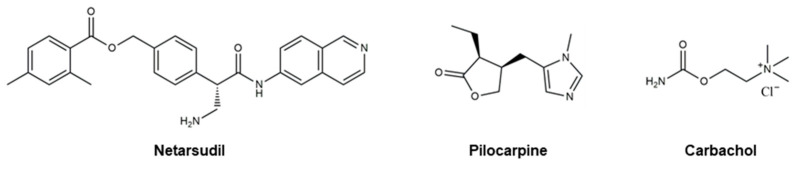
Chemical structures of a Rho kinase inhibitor, **netarsudil**, and two cholinergic agents, **pilocarpine** and **carbachol**, with reported clinical efficacy in managing intraocular pressure (IOP) for the treatment of glaucoma.

Even though there are no cures for glaucoma currently available, research efforts have led to the development of effective measures for disease management. Based on the current understanding of glaucoma pathology, reduction in IOP is considered the most effective therapeutic approach to prevent its progression and further visual impairment. In POAG patients, IOP can be reduced through eyedrops, laser therapy, and surgical interventions. Every 1 mmHg of IOP-lowering has been associated with an estimated 10–19% reduction in the risk of progression in patients with glaucoma [107,108,109]. A target IOP is determined based on structural and functional disease progression and the recorded IOP under pathological conditions [82]. Experts denote the importance of considering the substantial interindividual variability of glaucoma, arising from differences in ocular anatomy, IOP-sensitivity of the optic nerve head, and onset of vision loss. Glaucoma patients are often prescribed treatments that are less invasive first. When insufficient reductions in IOP are observed after a designated period of treatment, more invasive approaches, such as laser and surgical procedures, are utilized. Various ophthalmic formulations in the form of eyedrops have been developed as glaucoma therapeutics, employing active pharmaceutical ingredients (APIs) such as prostaglandin analogs, beta blockers, alpha agonists, carbonic anhydrase inhibitors, Rho kinase inhibitors, and cholinergic (miotic) agents (Table 2). In this section, these groups of glaucoma therapeutics are discussed regarding their chemical structures, pharmacology, mechanism of action (MoA), efficacy in IOP reduction, and side effects. The molecular structures of glaucoma therapeutic compounds are presented in Figure 3, Figure 4, Figure 5, Figure 6 and Figure 7.

### 4.1. Prostaglandin Analogs

Prostaglandins are hormone-like lipids involved in various biological functions: inflammation regulation, smooth muscle contraction, platelet aggregation, and blood flow regulation [110]. With respect to glaucoma treatment, prostaglandin analogs (PAs) aim to mimic the actions of prostaglandins in two ways: (i) binding to the prostaglandin F2α (FP) receptors in the ciliary muscle, increasing tissue permeability, and increasing the uveoscleral outflow of aqueous humor; and (ii) binding to prostaglandin receptors in the ciliary epithelium, responsible for producing aqueous humor, and inhibiting the production and secretion of aqueous humor [111]. The dualistic approach of PAs in mitigating the intraocular accumulation of aqueous humor renders notable efficacy in reducing IOP. The exact underlying mechanisms of PAs are not yet fully understood, but research indicates the regulation of matrix metalloproteinase (MMP) and remodeling of the extracellular matrix, altering the outflow pathways, contributes to deviations in outflow resistance [111]. Furthermore, the ocular hypotensive effects of PAs have been suggested to be a result of their action on prostanoid receptors and signal-transduction systems such as intracellular Ca(II) and cyclic AMP [112]. PAs are considered to be well-tolerated in glaucoma patients relative to other glaucoma therapeutic APIs. However, they exhibit several side effects that range from minor to more severe. PAs can induce eye irritation and discomfort, increased iris pigmentation, increased length and thickness of eyelashes, skin darkening around the eye, conjunctival hyperemia (redness), and periorbital fat loss. Recognized for its therapeutic efficacy and minor side effects, prostaglandin analogs are often employed as the first prescription eyedrops in new glaucoma patients. Despite its effectiveness, clinical use over the years has revealed the existence of patients who do not respond well to prostaglandin F2α derivatives, denoting the necessity for adjunctive therapy or alternative therapeutics in those cases [113,114]. Molecular structures of the currently available PAs are presented in Figure 3.

Approved for medical use in the U.S. in 1996, **Latanoprost** (Figure 3) is an ester prostaglandin F2α analog sold under the brand name Xalatan^®^ (Pfizer, Inc., New York, NY, USA) [115]. Topical formulation of **latanoprost** (0.005%) is used to treat ocular hypertension and POAG by reducing IOP. This PA continues to account for approximately 65% of PA prescriptions based on its excellent efficacy-tolerability profile [115]. It is worth noting that **latanoprost** generics were the first to be introduced among the prostaglandin analogs [116]. Improved formulations of **latanoprost** were later developed without benzalkonium chloride, a preservative used to improve stability and prevent contamination, to eliminate the associated ocular side effects [117]. Development of **latanoprost** involved the (i) esterification of the carboxylic acid functionality to improve corneal penetration and reduce side effects [118], and (ii) modification of the omega chain to enhance its selectivity for prostaglandin F receptors and tolerability profile [119]. Esterification of prostaglandin in **latanoprost** yields a more lipophilic compound, leading to greater absorption through the cornea, upon which the molecule is converted to **latanoprost** acid, the active metabolite, through hydrolysis. Following topical administration, peak [**latanoprost**] was observed after 1–2 h at circa 15–30 ng/mL in the aqueous humor [115]. The metabolite, **latanoprost** acid, was shown to be rapidly eliminated [120]. The liver is mainly responsible for the metabolism of **latanoprost** acid through the β-oxidation to 1,2-dinor and 1,2,3,4-tetranor latanoprost acid [120]. Reports indicate that IOP reduction can be detected within 3–4 h, exhibiting maximum effect between 8 and 12 h, and is maintained for a minimum of 24 h [121]. Multiple clinical studies concerning the efficacy and safety have demonstrated the notable IOP reduction efficacy and safety of short- and long-term topical **latanoprost** treatment [121,122,123,124,125]. **Latanoprost**-induced IOP reductions are time-dependent: application in the morning leads to a 31% reduction and evening application leads to a 35% reduction [122]. A UK glaucoma treatment study presented a significant reduction in visual field deterioration through daily **latanoprost** (0.005%) treatments when compared with placebo [126]. Minor side effects such as conjunctival hyperemia, iris darkening, and eyelash alterations were recounted from these studies. A 2015 study comparing the efficacy and safety of original and generic **latanoprost** stated that although their efficacies in reducing IOP were statistically similar, noticeable disparities were accounted for in the observed side effects [127].

**Bimatoprost** is a synthetic analog of prostamide F2α (Figure 3), introduced in 2001, that is sold under the brand name Lumigan^®^ (Allergan, Inc., Irvine, CA, USA) [128]. With more than three million prescriptions, **bimatoprost** was the 166th most prescribed medication in the U.S. Prostamides are a group of physiologically active lipid-like compounds that have similar chemical structures to prostaglandins. The molecular structure of **bimatoprost** manifests an amide functionality in place of the ester group on **latanoprost**. A second double bond is also present on **bimatoprost**. **Bimatoprost** is often employed when other therapeutic agents, such as **latanoprost**, are insufficient in effectively reducing IOP in glaucoma patients [128]. The exact MoA for prostamides remains convoluted; however, recent findings have demonstrated that **bimatoprost** dose-dependently upregulated MMP-1 and MMP-14 mRNA in all cell types, and MMP-10 and MMP-11 mRNA in the trabecular meshwork and ciliary muscle cells [129]. Despite the conspicuous structural similarities between PAs and prostamides, their MoAs are reported to be markedly different [130]. Furthermore, **bimatoprost** reportedly exhibits significant distinctions in pharmacokinetics (PK) from other Pas [131]. Unlike the conversion of **latanoprost** to latanoprost acid, the metabolic conversion of **bimatoprost** is minimal, indicating that **bimatoprost** is not a prodrug [132]. Unlike other PAs, **bimatoprost** does not act on the prostaglandin F receptor. Instead, studies indicate that **bimatoprost** mimics the activity of prostamide to create organized pathways for aqueous humor outflow in the ciliary body, providing the structural basis for increased uveoscleral outflow [128]. In addition, it produces marked increases in prostamide receptor-mediated hydraulic conductivity to promote the generation of trabecular meshwork/Schlemm’s canal outflow pathways [133]. A 2023 report comparing the cellular effects of **bimatoprost** and **bimatoprost** free acid further supports the idea that **bimatoprost**’s reductive effects on IOP stem from its prostamide-like activity on MMP expression and tissue remodeling [129]. Several studies indicate the superior efficacy of **bimatoprost** in reducing IOP relative to **latanoprost** with statistical significance [134,135]. After 6 months of comparative analysis in IOP measurements, mean IOP reductions were 1.2–2.2 mmHg larger in the **bimatoprost** group than in the **latanoprost** group [134,135]. **Bimatoprost** also provided significantly lower mean IOP at each follow-up time point and better diurnal IOP control than **latanoprost**. Furthermore, 83 to 89% of patients in the **bimatoprost** group compared with 65 to 72% in the **latanoprost** group responded to treatment with a 15% or greater IOP decrease at the 6-month diurnal IOP evaluation point. Concerning drug safety, research suggests that **bimatoprost** was tolerated to a degree comparable to that of **latanoprost** with the main side effects being conjunctival hyperemia.

**Travoprost** (Figure 3) is a synthetic ester prodrug of a prostaglandin F2α analog approved for medical use in the U.S. and EU in 2001. This topical agent is sold under the brand name Travatan^®^ (Novartis, Inc., Basel, Switzerland). In 2020, it was the 304th most prescribed medication in the U.S. with more than one million prescriptions. The chemical structure of **travoprost** presents a trifluoromethyl group at the meta-position of the benzyl functionality. The trifluoromethyl functionality is reported to (i) enhance the therapeutic efficacy of the molecule in reducing IOP by promoting receptor binding, (ii) increase the lipophilicity of the compound to enhance its ability to penetrate ocular tissues and facilitate its absorption through the cornea when applied topically, and (iii) enhance the bioavailability of the drug by making it more stable and less prone to enzymatic degradation [136,137]. Like **bimatoprost**, **travoprost** contains an additional double bond when compared to **latanoprost**. The metabolism of **travoprost** is analogous to that of **latanoprost**, where the isopropyl ester undergoes esterase-catalyzed hydrolysis to yield the biologically active free acid [138]. The resultant free acid is responsible for interacting with the prostaglandin FP receptors and inducing the downstream pathway, leading to increased uveoscleral and trabecular outflow of the aqueous humor and, ultimately, IOP reduction. In rabbits, a peak concentration of 20 ng/g of travoprost acid was detected in 1–2 h, exhibiting a half-life of 1.5 h [139]. Plasma concentrations of the free acid were measured at 25 ng/L at 10–30 min after administration and rapidly declined below the detection limit within 1 h [139]. Metabolism of travoprost acid takes place in a manner similar to that of **latanoprost** acid and other PAs: β-oxidation to 1,2-dinor and 1,2,3,4-tetranor travoprost acid [140]. In human subjects, the IOP reduction effects of **travoprost** could be detected within 2 h of topical administration. Maximum effect was reached in 12 h. The drug remained effective for a minimum of 24 h [140]. Four randomized, double-blind, multicenter, parallel-group studies found **travoprost** (0.004%) to be effective in reducing IOP as a monotherapy in POAG patients [141,142]. Topical administration of **travoprost** led to a mean reduction in IOP of circa 7 mm Hg, which was similar to that of **latanoprost** (0.005%) and superior to that of **timolol** (0.5%) [141]. Data indicate that utilizing **travoprost** as an adjunctive therapy with **timolol** exhibited greater efficacy in reducing IOP [138]. An interesting observation from these studies was the race-dependent effects of **travoprost** in reducing IOP [140]. Compared to latanoprost and **timolol**, **travoprost** exhibited superior therapeutic efficacy in Black patients. **Travoprost** was determined to be well tolerated by patients, with most adverse events being mild and moderate in severity, and resolving when treatment stopped [138,142]. Reported adverse events included conjunctival hyperemia, iris pigmentation, changes in eyelashes, blurred vision, pain, and discomfort [142].

**Tafluprost** (Figure 3), or Zioptan^®^ (Merck Inc., Rahway, NJ, USA), is a prodrug ester prostaglandin F2α-analog designed to expedite the corneal penetration of the drug, which is then hydrolyzed by corneal esterases to produce the carboxylic acid active metabolite. The product, tafluprost acid, can then be taken up by the aqueous humor to therapeutically relevant levels. As observed in **latanoprost**, esterification of the carboxylic acid group on the α-sidechain improves corneal penetration. As a selective agonist of the prostaglandin F receptor, tafluprost acid presents two fluorine atoms, on the β-chain, reported to enhance receptor binding [143]. The binding affinity of tafluprost acid for the prostaglandin F receptor was determined to be 126-fold greater than that for the prostaglandin E receptor 3 [112]. Pharmacokinetic analyses revealed that the prodrug could not be detected in any of the ocular tissue or plasma, while the carboxylic acid form was found in the cornea, aqueous humor, iris, and ciliary body 8 h after topical application [144]. No detectable systemic accumulation was observed 24 h post-dose. The abovementioned β-oxidation (see **latanoprost** section) is responsible for the degradation of the α-chain of **tafluprost** [112]. Three phase I studies have demonstrated the tolerability and safety of **tafluprost** [145]. A 2010 study demonstrated the superior IOP reductive properties of **tafluprost** in comparison to **latanoprost**, further evincing its tolerability [146]. A phase II study resulted in the establishment of the therapeutic concentration at 0.0015%, at which **tafluprost** displayed IOP reduction comparable to that of **latanoprost** 0.005% [147]. Various phase III investigations confirmed the noninferiority of the therapeutic efficacy of **tafluprost** relative to that of **latanoprost**, along with its effectiveness as an adjunctive therapeutic when paired with **timolol** [148]. Common side effects of **tafluprost** were moderate in severity, including those observed in other PAs, including iris hyperpigmentation, conjunctival hyperemia, discomfort, pain, photophobia, tired eyes, and blurred vision [143]. In vitro evaluation of **tafluprost**’s effect on melanin stimulation in melanoma cells demonstrated that the molecule did not noticeably alter cellular melanin levels, supporting its lower rate of iris and periocular pigmentation relative to **latanoprost** [147]. Photophobia and ocular hyperemia were more commonly reported with **tafluprost** compared to **latanoprost** [145]. A 2003 comparative study of **latanoprost**, **bimatoprost**, and **travoprost** reported an incidence rate of hyperemia of 5–20% with **latanoprost**, 35–50% with **travoprost**, and 35–50% with **bimatoprost** at 12 weeks [135]. Papadia et al. highlight the importance of minimizing the negative effects of antiglaucoma topical therapeutics to improve patient compliance [143].

**Latanoprostene bunod** (**LBN**; Figure 3) is a nitric oxide-donating PA used to reduce IOP that is commercially available as Vyzulta^®^ (Bausch & Lomb, Inc., Laval, Canada). This ophthalmic drug was approved for medical applications in the U.S. in 2017 as a means of IOP reduction in POAG and ocular hypertension patients. The molecular structure of **LBN** presents the same β-chain structure as **tafluprost** (Figure 3) with a difluoromethylene functionality. In addition, it consists of a nitric oxide-donating moiety, butanediol mononitrate, attached to the esterified α-chain. Rationally designed for a double-pronged strategy in reducing IOP, **LBN** is reported to be effective in patients who are unresponsive to other IOP reduction drugs [149]. **LBN** takes advantage of a dualistic MoA to increase aqueous humor outflow [107]. Carboxylic ester hydrolysis of **LBN** yields latanoprost acid and butanediol mononitrate. Latanoprost acid increases uveoscleral outflow, while the nitric oxide-donating moiety (butanediol mononitrate) releases nitric oxide to enhance outflow through the trabecular meshwork and Schlemm’s canal. The MoA for latanoprost acid is presented above in the **latanoprost** section. Nitric oxide plays a key role in blood regulation through its ability to relax vascular smooth muscle [150]. This aspect can be translated to the ocular effects of the molecule through the relaxation of the cells in the trabecular meshwork/Schlemm’s canal, resulting in increased outflow and lower IOP [151]. In vivo data indicate the reductive effects of nitric oxide on IOP through outflow augmentation are mediated by guanylate cyclase-1 and subsequent activation of the cyclic guanosine monophosphate (cGMP)/protein kinase G signaling cascade [151,152,153]. Furthermore, nitric oxide is reported to (i) act as a vasodilator to alter ocular blood flow and (ii) exhibit neuroprotective or neurodegenerative effects on the retinal ganglion cells of the optic nerve, depending on the concentration, nitric oxide source, and cell model [154,155,156,157]. Research regarding the possible neurodegenerative effects of nitric oxide on retinal ganglion cells suggests that although oxidative damage at high [nitric oxide] has been reported, its short half-life (circa <3 s) in extravascular tissue makes it improbable for **LBN**-associated nitric oxide from daily topical administration to reach such neurotoxic levels. Preclinical studies demonstrated significant IOP-lowering effects superior to that of **latanoprost** as a monotherapy [158]. The reductive effects of **LBN** on IOP through the delivery of nitric oxide were supported by studies utilizing (i) prostaglandin F receptor knock-out mice [159] and (ii) endothelin-1 contracted human trabecular meshwork cells [160]. Compared to **latanoprost**, **LBN** showed a more sustained effect in lowering IOP, further evincing the nitric oxide-mediated therapeutics effects of the compound. Numerous studies illustrate the clinical efficacy of **LBN**: (i) phase I study of 24 h IOP-lowering effects in healthy subjects [161], (ii) phase II dose-range-finding study against **latanoprost** 0.005% [162], and (iii) three phase III studies [163,164,165]. In the phase III studies, **latanoprost** 0.0024% once daily in the evening was determined to be more effective in lowering IOP than **Timolol** 0.5% administered twice daily with mean IOP being considerably lower at the majority of time points with statistical significance. These studies corroborated the non-inferiority and superiority of **LBN**’s therapeutic effects relative to **timolol**. An open-label study reported IOP reductions of 26.3% and 23.0% from the baseline with sustained consistency [166]. In a manner similar to other topical formulations of PAs, the most common ocular events were conjunctival hyperemia (5.9%), eye irritation (4.6%), and eye pain (3.6%) with moderate severity [165].

**Unoprostone** (Figure 3) is an isopropyl ester PA that was previously marketed as Rescula^®^ (Sucampo Pharmaceuticals, Inc., Rockville, MD, USA). It received FDA approval in 2000 for the treatment of POAG and ocular hypertension. In 2009, Sucampo Pharmaceuticals acquired the commercialization rights for the drug in the U.S. and Canada [167]. Several years later, the FDA revised its formal label to remove **unoprostone**’s characterization as a PA. The reason for this change is that unoprostone is a 22-carbon derivative of docosahexaenoic acid without biologically relevant binding affinity to the prostaglandin receptor, while PAs are 20-carbon derivatives of the eicosanoid prostaglandin F2α [168]. Despite the research efforts demonstrating the possible activation of potassium and chloride channels leading to the relaxing of the trabecular meshwork, Sucampo voluntarily discontinued the sale of Rescula and returned all licenses for the drug to R-Tech Ueno in 2015. Limited information is available regarding the reason for this discontinuation; however, a brief Google search leads to the conclusion that it was for business reasons rather than safety concerns. Unlike the other PAs illustrated in this review, the α-chain of unoprostone presents a carboxylic acid, while the α-chain consists of a decanone functionality. No descriptions regarding the rational design of the molecule could be found through a simple literature search. A risk-benefit assessment of latanoprost and unoprostone in 1999 concluded that unoprostone’s efficacy in reducing mean IOP was inferior to that of its counterpart, despite the fact that unoprostone was administered twice daily at a greater concentration (0.15% vs. 0.005%) [169]. In addition, research suggests that unoprostone-treated patients exhibited increased rates of adverse ocular effects including corneal epithelial keratopathy [169]. It is possible that these reasons made unoprostone less competitive compared to other topical agents, leading to the discontinuation of the therapeutic.

It should be noted that many glaucoma ophthalmic eyedrop formulations (e.g., PAs, beta blockers, alpha agonists, and carbonic anhydrase inhibitors) contain preservatives such as benzalkonium chloride, which is now understood to be a primary cause of ocular surface disorder (OSD) such as dry eye disease and blepharitis [143]. Benzalkonium chloride can be detected in the trabecular meshwork, corneal endothelium, lens, and retina following the topical administration of glaucoma ophthalmic solutions [170]. Furthermore, tissue accumulation of the compound can lead to corneal and conjunctival toxicity with considerable cellular consequences such as tight junction disruption, immunoinflammatory response, and apoptosis [170]. Of glaucoma patients, 25% were recounted to have complained of pain as an immediate side effect of topical instillation of glaucoma therapeutic eyedrops. Studies have demonstrated that eliminating benzalkonium chloride in ophthalmic formulations reduced the immediate burning sensations and discomfort associated with their topical administration [171]. Pain and burning sensations from the topical application of benzalkonium chloride are associated with tearing, which can lead to poor drug absorption through the cornea by the ’washout’ effect. Numerous studies indicate that adverse ocular effects from PAs can be significantly used through the application of preservative-free formulations [143]. 

### 4.2. Beta Blockers

Beta blockers (Figure 4) are a class of therapeutics that block the effects of adrenaline and similar hormones on beta-adrenergic receptors. They are systemically used to treat hypertension, cardiovascular disease, and glaucoma. By obstructing the beta-receptors of the sympathetic nerve endings in the non-pigmented ciliary epithelium in the eye, beta blockers diminish the production of aqueous humor to decrease IOP [172]. Two distinct types of beta blockers are available as glaucoma therapeutics: (i) nonselective beta blockers, which target both beta-1 and beta-2 adrenoreceptors, and (ii) cardioselective beta blockers that block only beta-1 receptors [173]. Nonselective beta blockers include **timolol**, **levobunolol**, **metipranolol**, and **carteolol**. **Betaxolol** is a cardioselective beta blocker that specifically targets beta-1 receptors. In addition to the receptor-mediated pathway, a vascular mechanism involving a decrease in the passive generation of aqueous humor by ultrafiltration has been reported [174]. Commercially available ocular beta blockers exhibit minimal systemic exposure; however, this aspect can be highly variable from patient to patient. Due to the potential systemic effects of beta blockers, healthcare providers are to take precautions when prescribing beta blockers for patients with preexisting heart or lung conditions. Two of the most detrimental adverse effects of beta blockers as glaucoma medication are the exacerbation of chronic obstructive airway disease with nonselective agents and the precipitation of bronchospasm.

**Timolol** (Figure 4) is a nonselective beta-adrenergic receptor-blocking agent. Commercial names for **timolol** include Betimol^®^ (Thea Pharma Inc, Lexington, MA, USA), Istalol^®^ (Bausch & Lomb, Tampa, FL, USA), and Timoptic^®^ (Merck Sharp & Dohme, Rahway, NJ, USA). With a morpholine thiadiazoyl modality presenting a butylamino propanol ether side chain, its chemical structure contains an asymmetric carbon atom and is provided as the levo-isomer [175]. Unlike PAs, **timolol** is not a prodrug and is delivered in its active form. **Timolol** exhibits strong interactions with beta-adrenergic receptors, with binding affinity reported in the low nanomolar range (circa 0.7 nM) [176]. Watanabe et al. have suggested that **timolol**’s effective reduction in aqueous humor production could be a result of its ability to decrease the blood flow to ciliary processes through its strong binding to the beta-adrenergic receptors [177]. Three concentrations of **timolol** are commercially available: 0.1, 0.25, and 0.5% [174]. The 0.1% is administered as a gel formulation, while the latter two are prepared as solutions. Two clinical studies evaluating **timolol**’s dose-dependent (0.1, 0.25, 0.5, 1.0, 1.5%) therapeutic efficacy concerning IOP reduction demonstrate its notable value as a glaucoma treatment [178]. In these studies, substantial decreases in IOP were observed in **timolol**-treated eyes, with its efficacy evident as early as 2 h after treatment, which continued up to 28 h post-treatment [178]. Similar levels of effectiveness were recorded for the 0.5% and 1.0% **timolol** solutions, reaching levels circa 40% below the baseline [178,179]. The 0.1% **timolol** gel employs carbomers (carbopol 974P), taking advantage of its mucoadhesive properties, to create a 3D network that can control a large quantity of water for prolonging ocular contact time and efficient delivery of the API. Due to the high viscosity of the pure carbomer that can cause blurred vision, a mixture of PVA and carbopol (TimoGel 0.1%) was developed [180]. TimoGel has been reported to improve (i) ocular bioavailability through efficient drug delivery that is often affected by the ‘washout’ effect from tearing and (ii) patient compliance in those experiencing dry eye disease. Preclinical studies show that the drug can be detected after 1 h of TimoGel topical instillation, while 0.5% **timolol** solutions exhibit diminished levels of the drug after 30 min [181]. Corneal penetration was 2.6–3.1-fold better with TimoGel relative to that of the 0.5% **timolol** solution. Such efficient drug delivery allowed researchers to achieve the same therapeutic efficacy while utilizing much lower concentrations of **timolol** (0.1% vs. 0.5%) [182]. Detailed reviews of the adverse effects of the topical glaucoma treatments containing **timolol** discuss the tolerability and safety of the drug [183,184]. Reported adverse effects of ocular **timolol** include skin pigmentation, discomfort, irritation, dry eyes, blurred vision, allergic reactions, and at times more serious systemic events such as low blood pressure, slowed heart rate, dizziness, shortness of breath, and fatigue. 

**Levobunolol** (Figure 4) is a nonselective beta blocker commercially available under trade names such as AKBeta^®^, Betagan^®^, and Vistagan^®^. As the L-enantiomer of bunolol, **levobunolol** is utilized as a hydrophilic HCl salt. The molecular structure of this molecule presents a dihydronaphthalenone modality accommodating a tert-butylamino propanol group linked via an ether bond. The ether chain of **levobunolol** is often observed in other beta blockers. Propranolol, another beta blocker used to treat hypertension and angina, and **levobunolol** exhibit noticeable structural similarities, with the latter possessing an unsaturated cyclohexanone. Research suggests the optimal corneal penetration of **levobunolol** with a log *p* value circa 2.4 [185]. In vivo studies indicate that the drug reaches anterior ocular tissues with corneal and iris C_max_ being reached 30 min after topical instillation. Primarily mediated by cytochrome P450 2D6, the oxidative metabolism of levobunolol yields dihydrobunolol, which manifests a half-life of circa 7 h [186]. As a major metabolite of **levobunolol**, dihydrobunolol reportedly exhibits equipotency toward the beta-adrenergic receptors [187]. This dualistic targeting of the beta-adrenergic receptors, by **levobunolol** itself and its metabolite, is potentially responsible for its extended duration of effect [188]. Reports evince the dose-dependent therapeutic efficacy of **levobunolol** in reducing IOP in healthy patients and those suffering from POAG and ocular hypertension [189]. Peak efficacy was demonstrated 2–6 h post-instillation with 1% **levobunolol** [189]. Effects of **levobunolol** were observed up to 12 h, indicating the potential necessity for twice daily administration for sufficient IOP management for a full 24 h period. **Levobunolol** 0.5% induced a 30% decrease in IOP in a 3-month clinical efficacy assessment study [190]. Of patients treated with **levobunolol** 0.5%, 72% experienced successful IOP control, while the success rate jumped to 79% with **levobunolol** 1% [191]. In a 15-month comparative analysis of **levobunolol** (0.5, 1%) and **timolol** (0.5%), the therapeutic impacts of both drugs were determined to be similar; IOP was reduced from the baseline 26–27 mmHg to 6.8–7.6 mmHg. Toxicity studies in animals have shown a large separation between the effective and toxic concentrations of **levobunolol**, implicating its physiological tolerability [188]. Ocular **levobunolol** treatment can lead to slight decreases in heart rate and blood pressure; such changes were, however, deemed clinically insignificant [189]. Adverse reactions from **levobunolol** treatment were reported in 5% of the subjects with the most significant events including the following: blepharitis, conjunctivitis, decreased visual acuity, superficial punctate keratitis, red eyes, itching, and burning [189,192]. 

**Carteolol** (Figure 4) is a nonselective beta blocker with intrinsic sympathomimetic activity used to treat glaucoma through IOP management [193]. Capable of binding to both beta-1 and beta-2 adrenergic receptors, **carteolol** has also been reported to show biologically relevant affinity towards serotonin 5-HT1A and 5-HT1B receptors [194]. Various commercial formulations of **carteolol** are available, including ocupress^®^, arteolol^®^, and glauteolol^®^. Presenting a dihydroquinolinone functionality, **carteolol** is equipped with a tert-butylamino propanol substituent, also found in other beta blockers such as **timolol** and **levobunolol**, connected via an ether group. Pharmacokinetic studies have demonstrated that **carteolol** is rapidly absorbed into the aqueous humor, reaching C_max_ 1 h post-administration [195]. **Carteolol** could be detected in the cornea, conjunctiva, and nictitating membrane after 1 h; and in the iris and ciliary body after 24 h. Cytochrome P450 2D6 is primarily responsible for metabolizing **carteolol** into 8-hydroxy-carteolol [196]. In vivo research showed that when ^14^C-carteolol was topically treated to one eye of a rabbit, the majority of the drug concentration in the treated eye remained as the original **carteolol**. Detectable levels of the active metabolite of **carteolol**, 8-hydroxy-carteolol, could also be detected in the control eye and plasma [193]. Reports indicate that circa 16% of **carteolol** is excreted in its original form via urine 24 h post-dose, with its urinary elimination half-life being approximately 5 h [193]. **Carteolol** 2% exhibited a terminal elimination half-life of circa 13.8 h [197]. The therapeutic efficacy of **carteolol** in reducing IOP is comprehensively reported. A crossover effect, where reduction in IOP can be observed in both eyes following topical treatment of only one eye, has been reported in some cases [198,199]. Watson et al. performed a 7-year comparative evaluation of the therapeutic efficacy of three beta blockers, **timolol**, **betaxolol**, and **carteolol** [200]. In this study, **carteolol** was determined to effectively reduce IOP (up to circa 30%) and maintain visual fields and acuity in POAG patients long-term [200]. In a phase IV study, where POAG patients were first treated with **latanoprost** for 8 weeks, a long-acting solution of **carteolol** 2% was shown to reduce IOP by 11.0% at Day 28 and 11.2% at Day 56 in POAG patients, to levels comparable to that of **timolol** 0.5% [201]. Hennes et al. provide a detailed account of studies assessing the clinical efficacy of **carteolol** [193]. A potential side effect of beta blockers is the reduction in ocular perfusion via local vasoconstriction, which can lead to decreased blood flow to the eye’s structures and potentially contribute to vision-related complications [193]. Unlike other beta blockers, **carteolol** exhibits intrinsic sympathomimetic activity, indicating its potential to instead increase ocular perfusion through its partial agonistic properties on the beta-adrenergic receptor [193]. Intrinsic sympathomimetic activity indicates partial agonism of a receptor that allows for the fractional stimulation of the beta-adrenergic receptor. This phenomenon is linked to the drug’s diminished impact on heart rate, blood pressure, and cardiac contractility. Studies suggest that the partial agonistic activity of **carteolol** is mediated by beta-2 adrenoreceptors in humans. [184] Common ocular side effects of beta blockers can also be observed from **carteolol**: discomfort, pain, shortness of breath, iris hyperpigmentation, conjunctival hyperemia, and overall asthenopia. More serious adverse events include bradycardia, systemic hypotension, and cardiac arrest [202].

**Betaxolol** (Figure 4) is a cardioselective beta blocker that can obstruct beta-1 adrenoreceptors for the treatment of glaucoma, hypertension, and angina. The ocular formulation of **betaxolol** is sold as Betopic^®^ (Novartis Inc., Basel, Switzerland). The drug’s relative selectivity for the beta-1:beta-2 adrenoreceptors was reported to be 245:1 [203]. This selectivity of **betaxolol** is often associated with a lower risk of systemic adverse effects, especially regarding its pulmonary implications. Patented in 1975, **betaxolol** was approved for medical use in 1985 for the treatment of glaucoma [204]. The molecular structure of **betaxolol** presents a benzene ring with two para-substituents in the form of side chains. Both chains consist of distinct ether functionalities: (i) isoproylamino propanol and (ii) ethoxymethyl cyclopropane. An interesting aspect of **betaxolol** and its drug formulation is the utilization of a racemic mixture, where the d-isomer is inactive and the l-isomer is known to be active [205]. A significant improvement in the therapeutic efficacy and further reduction in adverse effects may be achieved through the utilization of an isomerically pure formulation of the compound. In 1986, a comparative analysis of the clinical efficacy of **timolol** and **betaxolol** was performed [205]. This study demonstrated notable efficacy in reducing IOP at 0.125% and 0.25%. A 20% reduction in IOP was observed over 6 months. Among the 20 patient subjects, four patients were reported to not respond to **betaxolol** topical ocular treatment, whereas **timolol** was able to reduce the IOPs of all patients treated with the drug. Furthermore, the reductive effects of **timolol** on IOP were determined to be greater than that of **betaxolol** at the same concentration. In 1990, Weinreb et al. developed a 0.25% **betaxolol** suspension that exhibited the same level of therapeutic efficacy as its 0.5% **betaxolol** solution counterpart [206]. By utilizing this new formulation of **betaxolol** with a lower concentration, researchers were able to further reduce the instances of side effects and develop a safer topical agent for glaucoma patients with moderate chronic obstructive pulmonary disease and bronchial asthma [206]. Studies in the 1990s and 2000s have implicated the neuroprotective effects of **betaxolol**, capable of attenuating the excitotoxicity-induced damage to ganglion cells and the impact of ischemia on the retina [207]. Despite its less potent therapeutic efficacy in comparison to **timolol**, research indicates that **betaxolol** is a safer option for glaucoma patients with preexisting pulmonary or cardiovascular conditions such as asthma; however, caution is advised as the systemic effects of beta blockers are highly variable and unpredictable. 

**Metipranolol** (Figure 4) is a nonselective antiglaucoma beta blocker sold as OptiPranolol^®^, Betanol^®^, Disorat^®^, and Trimepranol^®^. The chemical structure of **metipranolol** presents a trimethylphenyl acetate functionality brandishing an isopropylamino propanol group linked via an ether bond. The side chain of **metipranolol** is equivalent to that of **betaxolol**. Limited information is available regarding the clinical pharmacokinetics of ocular **metipranolol**. Research indicates that oral **metipranolol** is rapidly absorbed, reaching plasma C_max_ within an hour post-treatment. Soon after, the drug is metabolized into deacetyl **metipranolol** [208]. The clinical efficacy of **metipranolol** has been demonstrated in numerous studies. First, ocular administration of a single drop of 0.6% **metipranolol** in healthy subjects resulted in a significant reduction in IOP (>3.5 mm Hg) 3 h post-therapy. Thereafter, the efficacy of a single dose of topical **metipranolol** 0.3% was studied in POAG patients. In this study, mean IOP decreases ranging from 31 to 38% from baseline were observed with the reductive effects of the dose persisting for up to 24 h and longer [209]. **Metipranolol** 0.1–0.6% demonstrated IOP reduction effects ranging from 20 to 29% decreases from baseline, determined to be comparable to the therapeutic activity of **timolol** 0.25–0.5% and **levobunolol** 0.5% [210,211]. In vivo studies regarding the unintended ocular effects of **metipranolol** have reported consequential corneal alterations: reduced number of microvilli covering the outer plasma membrane and widened intracellular spaces on the surface cell layers. These observations, however, were deemed to be insignificant considering the overall well-being of the eye [211]. At the clinical level, research endeavors monitored the hemodynamic effects of the ocular instillation of the drug, demonstrating that systolic or diastolic blood pressure and heart rate are not significantly altered following ocularly instilled **metipranolol** 0.1 to 0.6% [212]. Battershill et al. have provided an organized review of the clinical trials comparatively assessing the therapeutic efficacy of **metipranolol** with other beta blockers and antiglaucoma substances [211]. **Metipranolol** reportedly induces common ocular adverse effects that are observed with other beta blockers: stinging and burning in 12–56% of patients, hyperemia, reduction in tear production, and blurred vision [213]. 

### 4.3. Alpha Agonists

Alpha agonists (Figure 5) are a group of sympathomimetic compounds that selectively target alpha-adrenergic receptors. Among the two classes of adrenergic receptors, the alpha-receptors are excitatory, while the beta-receptors are inhibitory [214]. Alpha-adrenergic receptors are further divided into two subtypes: alpha-1 (A,B,D) and alpha-2 (A,B,C) [215]. Through their ability to activate Ca(II) channels or release intracellular Ca(II), alpha-1 receptors are capable of inducing the contraction of smooth muscle [215]. Oppositely, alpha-2 receptors are responsible for the inhibition of neurotransmitters [216]. Stimulation of post-junctional alpha-2 receptors in the vascular system results in vasoconstriction, whereas activation of post-junctional epithelial alpha-2 receptors leads to the inhibition of adenylate cyclase [217,218]. The ocular administration of alpha agonists is reported to result in the activation of alpha-1 and alpha-2 receptors. Activation of alpha-1 leads to the (i) contraction of the iris dilator and Muller’s muscles, leading to mydriasis and lid retraction, (ii) vasoconstriction causing the restriction of blood flow to the ciliary muscle, and (iii) reduced aqueous humor production [215]. Stimulation of vascular post-junctional alpha-2 receptors induces the vasoconstriction of the ciliary body and episcleral [218]. Moreover, the activation of the post-junctional epithelial alpha-2 receptors prompts a decrease in the production of aqueous humor and an increase in uveoscleral outflow by curtailing intracellular cAMP levels [216,219,220]. At the cellular level, alpha-2 receptor activation was reported to counteract the effects of beta-receptors on the aqueous outflow and mitotic activity in human trabecular meshwork endothelial cells [221]. Furthermore, alpha agonists have demonstrated the ability to influence the expression and enzymatic activity of MMPs and tissue inhibitors of metalloproteinase (TIMPs), factors that play a crucial role in regulating the degradation of the extracellular matrix (ECM), which can contribute to greater outflow resistance. Consequently, promoting the degradation of ECM could enhance aqueous outflow and support the long-term viability of a trabeculectomy bleb after surgery [222]. In the past, nonselective adrenergic agonists such as epinephrine and dipivefrin have been infrequently used to treat glaucoma or ocular hypertension [214]. These, however, have been largely replaced by alpha-2 selective agonists for reasons involving safety, tolerability, and efficacy [214]. 

**Apraclonidine** (Figure 5) is an alpha-2 adrenoceptor agonist and a weak alpha-1 agonist, sold under the brand name Iopidine^®^ (Novartis Inc., Basel, Switzerland). This alpha agonist was developed following clinical investigations regarding the systemic side effects of clonidine, which was previously used to reduce IOP in glaucoma and ocular hypotension patients. Studies found that optical administration of clonidine produced pronounced reductions in brachial systolic blood pressure. Hence, clonidine was replaced with other alpha agonists such as **apraclonidine**, which exhibited greater tolerability and safety. **Apraclonidine** is prescribed as a short-term adjunctive therapy for glaucoma patients already on maximally tolerated medical treatment, aiming to further reduce IOP [223]. The molecular structure of **apraclonidine** presents a 3,5-dichloroaniline functionality and a 4,5-dihydroimidazole moiety connected via a secondary amine. Limited information is available regarding the pharmacokinetics and pharmacodynamics of **apraclonidine**. Clinical studies have demonstrated the efficacy of **apraclonidine** monotherapy [224]. A multicenter, randomized, parallel, double-masked study revealed that **apraclonidine** (0.25%, 0.5%) topically administered three times a day effectively reduced IOP at week 2 to a magnitude comparable to that of **timolol** [225]. This reductive effect on IOP, however, was observed to dwindle after 30–90 days of treatment at the 0.25% concentration of **apraclonidine**. In contrast, the 0.5% **apraclonidine** group demonstrated a more persistent therapeutic effect from the eyedrops, inducing circa 20% decreases in IOP 8–12 h after administration, like **timolol**. The therapeutic efficacy of **apraclonidine** as an adjunctive therapy is notable as when the drug was topically administered in conjunction with **timolol** or **levobunolol**, an additional decrease in IOP ranging from 1.3 to 4.4 mmHg could be detected [226,227]. Side effects of **apraclonidine** include fatigue, drowsiness, dry mouth, dry nose, burning, conjunctival blanching, allergic reaction, eyelid retraction, and mydriasis. Allergic reactions have been reported during clinical trials [228]. 

**Brimonidine** (Figure 5) is a selective alpha-2 adrenoceptor agonist. Currently sold under the brand names Alphagan^®^ and Brymont^®^ (Allergan, Inc., Irvine, CA, USA), **brimonidine** was patented in 1972 and became commercially available as a glaucoma treatment as an eyedrop formulation containing **brimonidine** tartrate. According to the drug usage statistics, **brimonidine** was the 175^th^ most commonly prescribed medication in the U.S. with more than three million prescriptions in 2020. As a white to crystalline powder that is freely soluble in water, **brimonidine** presents a molecular structure resembling that of clonidine. Presenting a quinoxaline framework, the chemical structure of **brimonidine** manifests a secondary amine linker attached at the 3-position to a dihydroimidazole functionality. **Brimonidine** displays a greater selectivity towards alpha-2 adrenergic receptors relative to clonidine or **apraclonidine**, potentially due to its molecular structure and increased hydrophobicity. Research suggests that the corneal, conjunctival, and scleral pathways are the major routes for the intraocular absorption of **brimonidine** [229]. In a 2020 study, Suzuki et al. comparatively investigated the pharmacokinetics of **brimonidine** as a monotherapy and adjunctive therapy with **brinzolamide** in vivo [230]. In this study using rabbits, aqueous humor concentrations of **brimonidine** reached C_max_ (472 ± 359 ng/mL) at 1 h after topical administration (0.1%) and exhibited a rapid distribution in the retina/choroid [230]. The Tmax for 0.1% **brimonidine** was 1.5 h, the first time point, in the anterior and posterior retina/choroid. The fact that the first time point was determined to be the T_max_ and no earlier time points were available suggests that the T_max_ value here could be an overestimation; the actual pharmacokinetics of the compound could be more rapid than this quantitative data imply. The plasma concentration of **brimonidine** reached C_max_ (1420 ± 160 pg/mL) in 0.5 h and quickly decreased afterward [230]. Binding to ocular melanin reportedly can increase **brimonidine**’s absorption and retention based on its marked affinity for melanin-containing ocular tissues in vitro [231]. Clinical studies yielded dose-dependent C_max_ values < 0.3 μg/L after single-dose ocular administration of **brimonidine** 0.08, 0.2, or 0.5% in both eyes [232]. Extensive hepatic metabolism of **brimonidine** has been reported to generate up to 11 metabolites with liver aldehyde oxidase-catalyzed oxidation of the compound implicated as the major metabolic pathways in humans; products of this oxidative degradation include 2-oxobrimonidine, 3-oxobrimonidine, and 2,3-dioxobrimonidine [233]. Urinary excretion was responsible for a majority of **brimonidine** elimination (60–75%), while fecal excretion accounted for 15–35% [233]. The plasma elimination half-life of the alpha-2 agonist and its corresponding metabolites ranged from 2 to 5 h in healthy human subjects [231]. The therapeutic efficacy of **brimonidine** monotherapy has been demonstrated by five multicenter, randomized, double-masked, parallel-group studies [233]. In these studies, **brimonidine** was determined to effectively reduce IOP from baseline and compared with placebo at 0.08, 0.2, and 0.5% administered twice daily for 4–12 weeks [234,235]. Comparative studies of **brimonidine** (0.2%) were performed against **betaxolol** (0.25%) and **timolol** (0.5%) [236,237]. IOP reductive effects of **brimonidine** were determined to be greater than that of **betaxolol** and similar to that of **timolol** under the clinical study conditions. **Brimonidine** was also able to slow down the progression of visual field loss to a greater degree with statistical significance when compared to the effects of **timolol** [238]. This study also revealed that a significantly greater proportion of the **brimonidine** group (55%) dropped out compared to the **timolol** group (29%). This observation implicates the greater potential for adverse reactions such as ocular allergies with the repeated ocular administration of **brimonidine** [238]. The most common side effects of **brimonidine** were hyperemia (26.3%), burning/stinging (24.0%), blurred vision (17.5%), and foreign body sensation (17.0%) [239]. Other ocular adverse events were reported with an incidence of <11%, including pruritus, allergy, corneal staining and erosion, and conjunctival follicles [231]. Dry mouth was the most prevalent systemic adverse effect with **brimonidine** 0.2% topical administration, with a mean incidence rate of 23.4% [231]. Others included headache, fatigue, and drowsiness. In a small number of cases, minor changes in systolic and diastolic blood pressure and heart rate have been reported from topical **brimonidine** [239]. 

### 4.4. Carbonic Anhydrase Inhibitors

Carbonic anhydrases are a classification of zinc-containing metalloenzymes that catalyze the interconversion between CO_2_ and H_2_O and carbonic acid, a key chemical reaction in the regulation of blood pH. This reaction is also a key process in the production of aqueous humor by the ciliary body as sodium bicarbonate is a main constituent of the aqueous humor. Furthermore, the bicarbonate ion affects the sodium dynamics of the eye and the homeostasis of water, ultimately influencing aqueous humor production [240]. Sixteen carbonic anhydrase isoforms can be found in humans with varying cellular/histochemical localization and enzymatic properties [241]. By suppressing the activity of carbonic anhydrase, the production of aqueous humor can be restricted, ultimately leading to a decrease in IOP. More specifically, carbonic anhydrase isoforms II, IV, and XII have been indicated to be responsible for the ocular bicarbonate secretion obtained as a product of the catalyzed hydration of CO_2_. Based on these notions, carbonic anhydrase inhibitors (CAIs; Figure 6) have been used to control IOP in glaucoma patients [241]. It should be noted that patients with G6PD deficiencies should be prescribed CAIs with caution due to the possible adverse reactions associated with sulfonamide derivatives [242]. In this section, four CAIs will be discussed: two topical treatments (**dorzolamide** and **brinzolamide**) and two oral formulations (**acetazolamide** and **methazolamide**). Both **acetazolamide** and **methazolamide** are considered first-generation CAIs used as systemic drugs for the management of glaucoma, while **dorzolamide** and **brinzolamide** represent the second-generation CAIs that are available as topical formulations [243]. 

**Acetazolamide** (Figure 6) is a heterocyclic sulfonamide exhibiting strong inhibitory effects (low nanomolar range) against a majority of the carbonic anhydrase isoforms [244]. Sold under the brand name Diamox^®^ (Teva Pharmaceuticals Inc., Tel Aviv-Yafo, Israel), **acetazolamide** (125 and 250 mg) is commercially available as an oral tablet for systemic administration. It is listed on the World Health Organization’s list of essential medicines and is available as a generic medication. The chemical structure of **acetazolamide** presents a central 1,3,4-thiadiazole framework with two adjunct substituents: acetamide and sulfonamide. Research indicates notable patient variability in the gastrointestinal absorption of oral **acetazolamide**, potentially indicating that the drug could benefit from being administered on a fasting stomach [245]. Peak plasma Tmax for an oral dose of 500 mg **acetazolamide** was circa 1.25–3 h and plasma half-life ranged from 2.4 to 5.8 h. The pharmacokinetics of oral **acetazolamide** was reviewed by Lehmann et al. [245]. The therapeutic efficacy of **acetazolamide** is supported by a number of clinical studies [246]. Orally administered **acetazolamide** was observed to reduce IOP within the first hour, demonstrating a 50–60% reduction in pressure [246]. These reductive effects were reported to diminish around the 8 h mark. The most effective reduction in intraocular pressure (IOP) is reportedly achieved with a regimen of 250 mg tablets taken four times daily or 500 mg sustained-release capsules administered twice daily. The degree of IOP reduction typically ranges between 20% and 30%, depending on the initial IOP levels [240]. The oral administration of **acetazolamide** prompts the systemic exposure of the drug, which could lead to more problematic adverse reactions compared to topical formulations. Anorexia, depression, nephrolithiasis, renal failure, malaise, weight loss, and paresthesia are among the systemic extraocular side effects of oral **acetazolamide** [245]. For these reasons, oral glaucoma therapeutics have been largely replaced by topical formulations and **acetazolamide** is utilized as an adjunctive therapy for patients experiencing inadequate IOP reduction through topical agents.

**Methazolamide** (Figure 6) is a carbonic anhydrase inhibitor sold under the brand name Neptazane^®^ (Perrigo Inc., Grand Rapids, United States) for the treatment of POAG and secondary glaucoma. Its utilization in glaucoma treatments has recently been experiencing a downward trend due to the systemic and localized adverse effects associated with its oral administration. The chemical structure of **methazolamide** resembles that of **acetazolamide**, presenting the same major framework as the thiadiazole brandishing acetamide and sulfonamide moieties. The key distinguishing factor of **methazolamide** from **acetazolamide** is the methyl group on the nitrogen atom of the thiadiazole and the change in resonance accompanying the tertiary amine group. This minor change in structure has been associated with (i) a greater inhibitory activity against carbonic anhydrases in vitro and (ii) improved delivery of the compound to the aqueous humor and spinal fluid from the plasma [247]. A major distinction between **methazolamide** and **acetazolamide** is reportedly their pharmacokinetics; **methazolamide** is shown to be more effective in diffusing into tissues and fluids due to its enhanced lipid solubility and low plasma protein binding [248]. Maren et al. investigated the pharmacology and inhibition of carbonic anhydrase by both **methazolamide** and **acetazolamide** in 1977 [248]. The plasma half-life of oral **methazolamide** was 14 h, while that of **acetazolamide** was 5 h [248]. Becker presented a comparative study of the clinical efficacy of **methazolamide** and **acetazolamide** in 1960 [249]. Herein, **methazolamide** reportedly could more effectively lower IOP, relative to **acetazolamide**, based on its improved pharmacokinetics. It was able to yield IOP reduction comparable to **acetazolamide** at concentrations four-fold smaller than its counterpart. Furthermore, these lower doses of **methazolamide** resulted in less renal and systemic effects such as plasma bicarbonate reduction and systemic acidosis [250]. More recent studies have indicated serious ocular and systemic side effects from oral **methazolamide**. In an interventional case report of a 70-year-old male, the patient developed bilateral acute myopia and PACG after ingesting **methazolamide** tablets for the treatment of normal tension glaucoma [251]. Although such observations are rare, the association between several sulfonamide-derived medications and secondary myopia and angle closure raises alarms for the utilization of the chemical in treating glaucoma. Numerous efforts have been made to improve the pharmacokinetics of **methazolamide** as a topical agent through the utilization of various technologies, including nanoparticles. Such endeavors, however, have yet to yield a commercially available product [252,253].

**Dorzolamide** (Figure 6) is a highly selective inhibitor of carbonic anhydrase II, sold under the brand name Trusopt^®^ (Merck Inc., Rahway, NJ, USA). Often used in combinational therapy with **timolol** and brimonidine, **dorzolamide** was approved for medical applications in 1994 in the U.S. Currently available as a generic medication, it was the 216th most prescribed medication in the U.S., with more than two million prescriptions. This second-generation carbonic anhydrase inhibitor is a heteroarene presenting a heterocyclic framework combining a thiopyran and thiophene [254]. The thiopyran presents dioxide, ethylamine, and methyl substituents, while the thiophene group is directly linked to a sulfonamide moiety. In vivo, research suggests that **dorzolamide** readily permeates into ocular tissue and fluid [255,256]. Balfour et al. report that **dorzolamide** concentrations greater than 5 mg/kg can be detected in the cornea, iris/ciliary body, retina, and aqueous humor after topical instillation at 2% [256]. Systemic exposure to **dorzolamide** from topical treatment can take place by drainage through the nasolacrimal duct and absorption from the nasopharyngeal mucosa [256]. Upon binding to carbonic anhydrase, **dorzolamide** can be metabolized by cytochrome P450 into an active metabolite, *N*-desethyldorzolamide, which can accumulate in erythrocytes [255]. Erythrocyte concentrations of **dorzolamide** achieved a stable level around 8 days of treatment with **dorzolamide** 2% eyedrops applied three times daily to both eyes of a healthy volunteer [256]. In a group of 56 patients diagnosed with glaucoma or ocular hypertension, the mean concentrations of **dorzolamide** and its metabolite in red blood cells were measured at 20.5 and 7.7 µM, respectively, following 12 months of treatment with **dorzolamide** 2% eyedrops applied three times daily [257]. Plasma concentrations of **dorzolamide** were found to be 11 µg/L after six months of treatment [255]. Approximately 33% of **dorzolamide** was found to be bound to plasma proteins. Topical **dorzolamide** is reported to have a half-life greater than 120 days and is mainly excreted in its original form in urine. Formulated as a 2% eyedrop to manage glaucoma and ocular hypertension, **dorzolamide** shows a 4000-fold greater affinity for carbonic anhydrase II than carbonic anhydrase I [256]. The inhibitory activity of **dorzolamide** against carbonic anhydrase II was reported to be significantly more potent than **acetazolamide** and **methazolamide**, the oral carbonic anhydrase inhibitors presented above [256]. Inhibiting carbonic anhydrase II in the ciliary processes of the eye decreases bicarbonate production, leading to a subsequent reduction in the production of aqueous humor and a decrease in IOP [256]. Twelve months of topical treatment with 2% **dorzolamide** resulted in an average carbonic anhydrase II activity reduction of 12% in the peripheral red blood cells of POAG patients. Clinical studies report that **dorzolamide** can effectively manage glaucoma and ocular hypertension through topical administration to the eye three times daily. With a mean IOP reduction of circa 4–6 mmHg 2 h post-dose and circa 3–4.5 mmHg 8 h post-dose, the therapeutic efficacy of 2% **dorzolamide** was deemed comparable to that of 0.5% **betaxolol** twice daily, and slightly inferior to that of **timolol** 0.5% [258]. Balfour et al. highlighted the effectiveness of **dorzolamide** as an adjunctive therapy to a degree similar to 2% **pilocarpine** [256]. Relative to the oral formulations of carbonic anhydrase inhibitors, **dorzolamide** eyedrops were substantially more effective in reducing IOP. As a topical formulation, **dorzolamide** exhibits significantly reduced systemic effects. The therapeutic compound is associated with bitter taste, transient local burning or stinging, digestive disturbances, headaches, blurred vision, itching, tearing, foreign body sensation, eyelid discomfort, and conjunctivitis [256]. Although rare, more serious adverse effects such as cardiovascular effects (angina, hypertension, tachycardia) and irreversible corneal decompensation have also been documented [259,260,261].

**Brinzolamide** (Figure 6) is a highly specific, non-competitive, and reversible carbonic anhydrase II inhibitor sold under the trade name Azopt^®^ (1% **brinzolamide** topical formulation; Novartis Inc., Basel, Swtizerland). Available as a generic medication in the U.S. since 2020, **brinzolamide** is a white powder commercially formulated as a 1% ophthalmic suspension used to reduce IOP in the treatment of POAG and ocular hypertension [262]. The molecular structure of **brinzolamide** exhibits notable similarities with that of **acetazolamide**. In **brinzolamide**, the *N*-methyl functionality of **acetazolamide** is replaced with a methoxy propane group. This structural distinction makes **brinzolamide** more lipophilic and reduces its aqueous solubility relative to **dorzolamide** or **acetazolamide** at physiological pH. As a result, **brinzolamide** can be prepared as a more comfortable eye suspension at pH 7.4 compared to the acidic pH of the **dorzolamide** solution (pH 5.6) [263]. The binding affinity for carbonic anhydrase II of **brinzolamide** is circa four-fold greater than that of **dorzolamide** [263]. Limited information is available regarding the pharmacokinetics of **brinzolamide**. **Brinzolamide** 1% is readily absorbed into the conjunctiva, cornea, iris, ciliary body, aqueous humor, lens, choroid, and retina. Peak concentrations of the compound are reached after 0.5–2 h of topical administration. Following topical instillation, **brinzolamide** enters the blood circulation via drainage through the nasolacrimal duct and absorption at the nasopharyngeal mucosa. An in vitro study conducted in human plasma has reported concentration-independent plasma protein binding of **brinzolamide**, falling within the range of 59% to 63% [263]. The primary metabolic breakdown of **brinzolamide** occurs mainly within the liver through oxidative *O*- and *N*-dealkylation processes mediated by cytochrome P450 enzymes. In erythrocytes, the predominant metabolite is *N*-desethyl-brinzolamide, which exhibits strong binding to CA-I. This metabolite is notably present in whole human blood but not in plasma. Minor metabolites, namely *N*-desmethoxypropyl-brinzolamide and *O*-desmethyl-brinzolamide, have been identified in urine but are not typically found in human whole blood. The majority of **brinzolamide** is excreted in the urine, with approximately 60% of it being eliminated unchanged and an additional 20% as the N-desethyl metabolite. Notably, **brinzolamide** exhibits a prolonged half-life in whole blood, as demonstrated by a half-life of 111 days after the topical administration of **brinzolamide** 3% ophthalmic suspension three times daily for 14 days in a cohort of 15 healthy male volunteers. Several clinical studies demonstrate the therapeutic efficacy of **brinzolamide** in reducing IOP to treat glaucoma and ocular hypertension. In comparison with **dorzolamide**, 1% **brinzolamide** exhibited the ability to significantly reduce IOP as a monotherapy to a degree similar to 2% **dorzolamide**, but less than 0.5% **timolol**. The results of these studies proved 1% **brinzolamide** to be the optimal concentration for IOP reduction through twice daily topical instillations. The short-term efficacy of **brinzolamide** 1% was shown by mean IOP reductions ranging from 13.2 to 21.8% through twice and three-times daily administration [264]. Following three months of treatment in two separate monotherapy trials, **brinzolamide** 1% administered either twice daily or three times daily resulted in an IOP response, defined as a reduction in IOP of at least 5 mmHg, or the maintenance of IOP control (IOP not exceeding 21 mmHg) in approximately 75.7% and 80.1% of patients, respectively. Systemic adverse effects are rare from this topical formulation of **brinzolamide** because of the low systemic levels of the drug that lead to incomplete saturation and inhibition of carbonic anhydrase II in erythrocytes and kidneys. **Brinzolamide**’s low affinity towards other isoforms of carbonic anhydrase further decreases the risk of systemic reactions [265]. It is possibly for these reasons that reports indicate the relatively good tolerability of **brinzolamide** 1% ophthalmic suspension. In long-term (18-month) clinical trials, the most common ocular adverse events related to the drug were temporary blurring of vision and ocular discomfort (i.e., stinging and burning ocular sensations) [265]. In certain cases, bitter or sour tastes have been reported [263]. In a 2000 study, researchers claimed that the switch from **dorzolamide** to **brinzolamide** resulted in overall improvements in comfort and ocular hypotensive efficacy, which further denotes the value of **brinzolamide** as a glaucoma therapeutic agent [266]. 

### 4.5. Rho Kinase Inhibitors

Rho kinases (ROCK) are a family of serine-threonine-specific protein kinases involved in regulating the shape, motility, proliferation, and apoptosis of cells through their actions on the cytoskeleton [267,268]. The Rho kinase family consists of three small guanosine triphosphate (GTP)-binding proteins (RhoA, RhoB, RhoC) [267]. Upon activation through GTP binding, Rho initiates the activation of its effector molecules, notably Rho kinase (ROCK1 and 2), which subsequently transmit signals to downstream molecules, inducing the polymerization of actin fibers [269]. This process occurs in various physiological systems, including the cardiovascular, pulmonary, and renal systems. In 2001, the role of Rho GTPases in aqueous humor outflow was initially proposed due to their presence in the trabecular meshwork, where they could induce calcium sensitization, leading to smooth muscle contraction in rabbit eyes [270,271]. Subsequent investigations revealed notably increased levels of RhoA, identified through immunostaining, in the optic nerve head of glaucomatous eyes compared to age-matched control eyes [272]. This finding underscores the connection between Rho proteins and the pathophysiology of glaucoma. Previous studies have indicated that ROCK and Rho GTPase inhibitors can increase aqueous humor drainage in the trabecular meshwork [273,274]. This phenomenon is a result of the ROCK inhibitor-induced reversible modifications to cell morphology and cell interactions in the eye, including the broadening of the extracellular space and juxtacanalicular tissue and actomyosin cytoskeletal organization [274]. Currently, there are two commercially available Rho kinase inhibitors: (i) **netarsudil**, approved in the U.S. and (ii) ripasudil, approved in Japan and China. In this section, the molecular structure, pharmacology, therapeutic efficacy, and safety/tolerability of **netarsudil** will be briefly discussed.

**Netarsudil** (Figure 7) is a ROCK inhibitor developed by Aerie-Pharmaceuticals Inc. and sold under the brand name Rhopressa^®^. With its FDA approval in 2017, **netarsudil** 0.02% was a first-in-class glaucoma therapeutic used as a once daily eyedrop. The chemical structure of **netarsudil** manifests an amino-isoquinoline amide, in which an aminoethylbenzyl 2,4-dimethylbenzoate is attached to the isoquinoline amide moiety via a formamide linker. The inhibitory activity of **netarsudil** against ROCK1 and ROCK2 is in the low nanomolar range [275]. Such inhibition of the kinases was observed to result in disturbances in the actin stress fibers and focal adhesions in trabecular meshwork cells. In addition to its inhibitory properties against ROCK, **netarsudil** can also inhibit norepinephrine transporter (NET). The inhibition of NET has recently been linked to a decrease in aqueous humor production [276]. In conjunction, the inhibitory activity of **netarsudil** against ROCK and NET presents a dualistic mechanism of action against glaucoma pathology by increasing aqueous humor outflow and decreasing aqueous humor production [276]. Pharmacokinetics investigations revealed that a single topical ocular dose of **netarsudil** 0.02% led to the detection of the compound in the cornea, conjunctiva, iris/ciliary body, retina-choroid-plexus, aqueous humor, vitreous humor, and lens. The level of distribution also followed the order the ocular anatomical parts are presented. The analyzed ocular pharmacokinetics parameters of **netarsudil** are presented in detail by Lin et al. [277]. The authors stated that as expected for topical ocular dosing, maximum systemic concentrations of **netarsudil** in the blood, plasma, liver, and kidney were circa 200 to 3000-fold lower than those in the cornea and conjunctiva. As **netarsudil** passes through the cornea into the aqueous humor, it is converted to a more potent molecule, **netarsudil**-M1, by corneal esterases. This converted form of **netarsudil** was suggested to be the predominant form in the aqueous humor following topical ocular dosing [277]. Various clinical studies have demonstrated the therapeutic efficacy of the ophthalmic formulation of **netarsudil**: (i) 0.02% **netarsudil** was reported to increase the mean diurnal outflow by 0.22% after 7 days of treatment [276], (ii) once daily dosing of **netarsudil** 0.02% reduced the mean diurnal IOP by 5.7–6.8 mmHg [278], (iii) noninferior clinical efficacy in reducing IOP, relative to **timolol**, in two separate phase 3 trials [279], and (iv) long-term efficacy in consistently lowering IOP throughout a 12-month study period [280]. The larger clinical studies have also monitored and investigated the tolerability and safety of the ROCK inhibitor. The most commonly reported adverse events following the topical instillation of **netarsudil** were related to the eye. The most frequent ocular side effect was conjunctival hyperemia, occurring at rates of 61% from once daily treatments. The following most prevalent adverse reactions were (i) corneal deposits, specifically corneal verticillata, with an incidence of 26%; and (ii) conjunctival hemorrhage, typically petechial, with an incidence of 20%. The abovementioned side effects were generally categorized as mild, with conjunctival hyperemia and/or hemorrhage occurring sporadically [280]. 

### 4.6. Cholinergic (Miotic) Agents

Introduced in 1877, cholinergic agents were the first class of ocular hypotensive compounds employed for the treatment of glaucoma [281]. Through direct and indirect action on the ocular muscarinic receptors, cholinergic agents induce pupil constriction and ciliary muscle contraction [281]. Such actions lead to the broadening of the trabecular meshwork. The subsequent increase in aqueous humor outflow is responsible for the reduction in IOP. Based on the critical role of the cholinergic systems in visual function, cholinergic agents such as **carbachol** (Isopto Carbachol^®^) and **pilocarpine** (Isopto Carpine^®^) have been used to treat and manage glaucoma. Due to the adverse effects associated with nonselective muscarinic receptor activation, these medications are usually not utilized as first-line treatments against glaucoma, but as adjunctive therapies in combination with other more effective therapeutics when inadequate IOP reduction is observed in patients. Limited information is available regarding the pharmacology and clinical efficacy of this class of glaucoma medications.

**Pilocarpine** (Figure 7) is an alkaloid miotic exhibiting constrictive effects on the pupil. As a muscarinic acetylcholine agonist, the alkaloid is utilized in the treatment of PACG and acute angle closure glaucoma as an ophthalmologic eyedrop. The molecular structure of **pilocarpine** presents an ethyldihydrofuranone attached to a methylimidazole via a methylene linker. Limited information is available regarding its ocular pharmacological properties. In 2019, See et al. reported the pharmacokinetics and tissue distribution of topically instilled **pilocarpine** in vivo [282]. In this study, the authors aimed to comparatively evaluate the pharmacokinetics of two delivery methods for the drug: topical eyedrop instillation vs. topical eyelid application. The concentrations of the **pilocarpine** solution used in this study, however, are not equal. Their results showed that the topical instillation of the eyedrops resulted in rapid drug penetration into the eye by absorption across the cornea from the precorneal tear film [282]. Fast elimination of the drug at the application site was also reported, noticeably affecting the drug delivery efficiency of the eyedrop formulation [282]. These results highlighted the limitations manifested by the washout effect caused by tearing that could result in increased systemic exposure through the absorption of the drug through the nasolacrimal duct drainage [282]. **Pilocarpine**’s effect on the muscarinic receptors in the ciliary muscle leads to contraction and traction at the scleral spur that can reduce the outflow resistance of aqueous humor through the trabecular meshwork and Schlemm’s canal [281]. It should be noted that **pilocarpine** can agonize M1-3 receptor subtypes. The M3 receptor, an excitatory receptor expressed in smooth muscle cells in the pupillary sphincter and ciliary bodies, can activate the Gq receptor and, in turn, phospholipase C, leading to the production of the second messenger’s inositol triphosphate and diacylglycerol, and calcium and protein kinase [283]. Ultimately, this **pilocarpine**-induced biochemical signaling results in the contraction of the pupillary sphincter muscle, which causes the ciliary contraction, constriction of the iris, and increased aqueous humor outflow [284]. Skaat et al. reported the clinical efficacy of the topical administration of **pilocarpine** 1% in the clinical expansion of Schlemm’s canal with (24%) and without glaucoma (21%) [285]. No direct comparisons were made between the spatial expansion of Schlemm’s canal and changes in the IOP. The dose-dependent IOP reductive effects of **pilocarpine** have been reported, demonstrating that concentrations above 8% did not yield greater IOP reduction [286]. Estimates suggest that topical instillation of **pilocarpine** can result in IOP reductions ranging from 20 to 30% [287]. Based on these notions, researchers have investigated the therapeutic potential of **pilocarpine** against PACG and acute angle closure glaucoma [288]. The exact therapeutic effects of **pilocarpine** in treating narrow-angle glaucoma, however, remain controversial.

**Carbachol** (Figure 7) is a synthetic cholinergic agent with greater potency relative to **pilocarpine**. The molecular structure of **carbachol** presents a positively charged choline carbamate encompassing a quarternary ammonium functionality. Limited literature is available regarding various aspects of this cholinergic agent. Approved for the treatment of glaucoma, **carbachol** is reported to exhibit its therapeutic effects through its actions on the muscarinic receptors and the inhibition of cholinesterases. The hydrophilic nature of **carbachol** elicits its poor absorption through the corneal epithelium following topical instillation. Therefore, benzalkonium chloride is often included in its eyedrop formulations to promote absorption, which is known to increase the incidence of ocular adverse reactions [289]. Despite a thorough literature screening focused on the ocular pharmacokinetics of **carbachol**, no such studies could be found.

### 4.7. Adjunctive Therapy

Adjunctive therapy in glaucoma treatment plays a pivotal role in enhancing the efficacy of managing intraocular pressure (IOP) and mitigating the progression of this sight-threatening disease. Glaucoma often necessitates multi-drug regimens to achieve target IOP levels, and adjunctive therapies provide additional means to attain this goal. These therapies can complement the actions of primary medications, ensuring a more comprehensive control over IOP. At present, PAs are often considered the first-line glaucoma therapeutics based on their success in controlling IOP and managing glaucoma exhibiting relatively mild side effects. Glaucoma pathology, however, presents substantial interindividual variability, with many patients, circa 30%, experiencing insufficient IOP regulation from PA monotherapy [290]. In such cases, adjunctive therapeutics (beta blockers, alpha agonists, carbonic anhydrase inhibitors, Rho kinase inhibitors, and cholinergic agents) are prescribed to attain adequate IOP reduction. Despite its benefits, it is crucial to consider potential drug-drug interactions when employing adjunctive therapies in combination with existing glaucoma medications. The careful selection and monitoring of adjunctive agents, along with a thorough understanding of their mechanisms and pharmacokinetics, are essential to maximize treatment benefits while minimizing the risks associated with interactions. Whitson published a review of adjunctive therapies against glaucoma [291]. Examples of widely prescribed fixed-combination glaucoma drops are Cosopt (**dorzolamide** and **timolol**), Combigan (**brimonidine** and **timolol**), Xalacom (**latanoprost** and **timolol**), Azarga (**brinzolamide** and **timolol**, Duotrav (**travoprost** and **timolol**), and Simbrinza (**brinzolamide** and **brimonidine**). **Timolol** is often included as an API in many of the fixed-combination therapeutics against glaucoma, further indicating the IOP reduction efficacy of the beta blocker for the management of glaucoma. In sum, adjunctive therapy is a valuable component of glaucoma management, enhancing both efficacy and patient tolerability when used judiciously and under proper clinical supervision. Recent innovations in the drug delivery systems for glaucoma therapeutics have led to the development of various topical formulations with enhanced pharmacological properties. Liposomes and nanospheres have been reported to provide enhancements in drug delivery efficiency, resulting in increased residence time of drugs such as pilocarpine [292]. These technologies aimed to increase the corneal exposure of the drug formulation, thereby effectively reducing dosing frequency. The utilization of contact lenses as a drug delivery vehicle has been garnering a great deal of attention based on its widespread clinical application. A pilot study demonstrated that contact lens-delivered timolol could effectively control IOP [293]. Furthermore, additional strategies for therapeutic glaucoma formulations are being explored to develop improved methods of IOP management such as sophisticated surgical implants and injectable systems [294].

## 5. Conclusions

As a complex and diverse group of eye diseases leading to irreversible vision loss, glaucoma presents a considerable threat to the vision health of the world population, especially with its aging. Substantial increases in average screen time may further contribute to the negative impact of glaucoma on society. Early detection of glaucoma is critical in preventing visual field loss; this matter is, however, challenging due to the stealthy nature of the disease, which lacks noticeable symptoms in its early stage. The emergence of AI-supported diagnostic tools promises a new frontier in early glaucoma detection, offering the potential for more timely and precise intervention. Such technological innovation, coupled with groundbreaking research in ocular drug delivery, gene therapy, and stem cell therapy, holds immense promise for vision recovery, particularly in late-stage glaucoma patients. These novel therapeutic strategies, aimed at targeting the underlying disease mechanisms and regenerating damaged ocular tissues, could revolutionize our approach to glaucoma management. Such advancements recount hope in (i) detecting glaucoma early, (ii) preserving vision through effective IOP management, and (iii) restoring vision in late-stage glaucoma cases through integrated, patient-centered approaches that harness the power of these emerging technologies. Through a detailed examination of glaucoma etiopathology, current diagnostic methods, and therapeutic molecules, this review highlights the significant advancements in the field, while also acknowledging the ongoing challenges in effectively managing and treating this condition. Tremendous advancements have been made in our ability to treat glaucoma, despite the challenges presented by the ocular barrier and associated side effects. With a greater understanding of glaucoma pathophysiology and ocular drug delivery, glaucoma therapeutics capable of protecting the vision of the aging world population may be within reach.

## Figures and Tables

**Table 1:** Various types of glaucoma and associated genes from previous research [6,7].

Types of Glaucoma	Associated Genes
Primary Open Angle Glaucoma (POAG)	*MYOC* (Myocilin) *OPTN* (Optineurin) *CYP1B1* (Cytochrome P450 1B1) *WDR36* (WD Repeat Domain 36) *TMCO1* (Transmembrane and Coiled-Coil Domains 1) *TXNRD2* (Thioredoxin Reductase 2) *TBK1* (TANK binding kinase 1)
Primary Angle Closure Glaucoma (PACG)	*ADAMTS10* (A Disintegrin and Metalloproteinase with Thrombospondin Motifs 10) *COL11A1* (Collagen Type XI Alpha 1 Chain) *PCMTD1* (Protein-L-Isoaspartate O-Methyltransferase Domain Containing 1) *GLC1N* (Primary Congenital Glaucoma 1N) *LOXL1* (Lysyl Oxidase-Like 1)
Normal Tension Glaucoma (NTG)	*CDKN2B-AS1* (CDKN2B Antisense RNA 1) *CAV1*/*CAV2* (Caveolin 1 and 2) *SIX1*/*SIX6* (Sine Oculis Homeobox Homolog 1 and 6) *TMEM136* (Transmembrane Protein 136) *ABCC5* (ATP Binding Cassette Subfamily C Member 5)
Secondary Glaucoma	*MYOC* (Myocilin) *CYP1B1* (Cytochrome P450 1B1) *TBK1* (TANK Binding Kinase 1) *ASB10* (Ankyrin Repeat and SOCS Box Containing 10) *FOXC1* (Forkhead Box C1)
Primary Congenital Glaucoma (PCG)	*CYP1B1* (Cytochrome P450 1B1) *LTBP2* (Latent Transforming Growth Factor Beta Binding Protein 2) *MYOC* (Myocilin) *CYP19A1* (Cytochrome P450 Family 19 Subfamily A Member 1) *LTBP2* (Latent Transforming Growth Factor Beta Binding Protein 2)
Pigmentary Glaucoma	*ADAMTS10* (A Disintegrin and Metalloproteinase with Thrombospondin Motifs 10) *OPA1* (Mitochondrial Dynamin Like GTPase) *TYR* (Tyrosinase) *TYRP1* (Tyrosinase-Related Protein 1) *MVP* (Major Vault Protein)
Exfoliative Glaucoma	*LOXL1* (Lysyl Oxidase-Like 1) *TGFBI* (Transforming Growth Factor Beta-Induced) *APOE* (Apolipoprotein E) *SRPX2* (Sushi Repeat Containing Protein X-Linked 2) *PEX11B* (Peroxisomal Biogenesis Factor 11 Beta)

**Table 2:** Therapeutic molecules for the treatment of glaucoma organized based on their classification and the trade names of the active pharmaceutical ingredients.

Classification	Active Pharmaceutical Ingredients
Prostaglandin Analogs	Latanoprost, Bimatoprost, Travoprost, Tafluprost, *Latanoprostene Bunod*, *Unoprostone*
Beta Blockers	Timolol, Levobunolol, Carteolol, Betaxolol, Metipranolol
Alpha Agonists	Apraclonidine, Brimonidine
Carbonic Anhydrase Inhibitors	Acetazolamide, Methazolamide, Dorzolamide, Brinzolamide
Rho Kinase Inhibitors	Netarsudil
Cholinergic (Miotic) Agents	Pilocarpine, Carbachol
Active Pharmaceutical Ingredients	Trade Names
Latanoprost	Xalatan, Xelpros, Monoprost,
Bimatoprost	Eyreida, Lumigan, Sturiban
Travoprost	Travatan, iDose TR, Travatan Z, Izba
Tafluprost	Taflotan, Zioptan, Saflutan
Latanoprostene Bunod	Vyzultar
Unoprostone	Rescula
Timolol	Betimol, Blocadren, Istalol, Timoptic, Timol
Levobunolol	AKBeta, Betagan, Vistagan
Carteolol	Arteolol, Arteoptic, Calte, Carteabak, Cateol, Cartrol, Elbloc, Endak, Glauteolol, Mikelan, Ocupress, Poenglaucol, Singlauc, Teopic
Betaxolol	Betoptic
Metipranolol	OptiPranolol, Betanol, Disorat, Trimepranol
Apraclonidine	Iopidine
Brimonidine	Alphagan, Mirvaso, Lumify, Brymont
Acetazolamide	Diamox
Methazolamide	Neptazane
Dorzolamide	Trusopt
Brinzolamide	Azopt
Netarsudil	Rhopressa
Pilocarpine	Diocarpine, Isopto carpine, Miocarpine, Pilopine HS
Carbachol	Miostat

## Data Availability

Data are contained within the manuscript.
